# Chimeric antigen receptor natural killer (CAR-NK) cell design and engineering for cancer therapy

**DOI:** 10.1186/s13045-021-01083-5

**Published:** 2021-05-01

**Authors:** Ying Gong, Roel G. J. Klein Wolterink, Jianxiang Wang, Gerard M. J. Bos, Wilfred T. V. Germeraad

**Affiliations:** 1 Division of Hematology, Department of Internal Medicine, Maastricht University Medical Center, Maastricht, The Netherlands; 2 GROW - School for Oncology and Developmental Biology, Maastricht University, Maastricht, The Netherlands; 3 Champalimaud Research, Champalimaud Centre for the Unknown, Lisbon, Portugal; 4 State Key Laboratory of Experimental Hematology, Institute of Hematology and Blood Diseases Hospital, Chinese Academy of Medical Sciences and Peking Union Medical College, 288 Nanjing Road, Tianjin, 300020 China; 5 National Clinical Research Center for Blood Diseases, Institute of Hematology and Blood Diseases Hospital, Chinese Academy of Medical Sciences and Peking Union Medical College, 288 Nanjing Road, Tianjin, 300020 China; 6 CiMaas BV, Maastricht, The Netherlands

**Keywords:** Cancer immunotherapy, Adoptive cell therapy, Chimeric antigen receptor, Natural killer cells, Genetic modification

## Abstract

Due to their efficient recognition and lysis of malignant cells, natural killer (NK) cells are considered as specialized immune cells that can be genetically modified to obtain capable effector cells for adoptive cellular treatment of cancer patients. However, biological and technical hurdles related to gene delivery into NK cells have dramatically restrained progress. Recent technological advancements, including improved cell expansion techniques, chimeric antigen receptors (CAR), CRISPR/Cas9 gene editing and enhanced viral transduction and electroporation, have endowed comprehensive generation and characterization of genetically modified NK cells. These promising developments assist scientists and physicians to design better applications of NK cells in clinical therapy. Notably, redirecting NK cells using CARs holds important promise for cancer immunotherapy. Various preclinical and a limited number of clinical studies using CAR-NK cells show promising results: efficient elimination of target cells without side effects, such as cytokine release syndrome and neurotoxicity which are seen in CAR-T therapies. In this review, we focus on the details of CAR-NK technology, including the design of efficient and safe CAR constructs and associated NK cell engineering techniques: the vehicles to deliver the CAR-containing transgene, detection methods for CARs, as well as NK cell sources and NK cell expansion. We summarize the current CAR-NK cell literature and include valuable lessons learned from the CAR-T cell field. This review also provides an outlook on how these approaches may transform current clinical products and protocols for cancer treatment.

**Supplementary Information:**

The online version contains supplementary material available at 10.1186/s13045-021-01083-5.

## Introduction

The human immune system has long been recognized as an important pillar of anticancer responses [1, 2]. As tumor cells originate from normal tissue, one of the main struggles in the design of anticancer therapies is the design of treatments that specifically target cancerous cells without affecting normal tissue. Ideally, these therapies have a strong potency against a particular malignancy that is characterized by rapid proliferation, resistance to apoptosis, and continuously changes through mutations and adaptations to the environment [3]. Recently, adoptive immune cell therapy (ACT) has shown promising clinical outcomes in patients with hematologic malignancies but remains difficult in treatment of solid tumors [4]. Unlike dispersed hematologic malignancies, solid tumors—especially late stage—exhibit an inhibitory tumor microenvironment due to hypoxia, a low pH, presence of suppressive cytokines, lactate, prostaglandins and others. These factors circumvent the ability of effector cells like NK cells to infiltrate and function in an optimal fashion [5]. Development of ACT should always be developed to overcome this induced tolerant state.

In ACT, cells are collected from autologous or allogenic donors and can be genetically engineered with a chimeric antigen receptor (CAR) that recognizing tumor-specific proteins on the cell surface. Often, this procedure also includes an ex vivo expansion step to obtain sufficient cell numbers [6, 7]. At this moment, the most successful form of ACT comprises of a CD19 CAR-T cell that cures up to 90% of patients with advanced refractory acute lymphoblastic leukemia (ALL). Here, complete remissions are attained within 4 weeks of treatment, as evident from several trials across multiple institutions [8, 9].

A CAR is composed of three domains: an ectodomain, the transmembrane region and a cytoplasmic activation tail [6, 10]. The ectodomain contains a single-chain variable fragment (scFv), usually derived from antibodies that provides the ability to specifically recognize tumor antigens expressed on cancerous cells [11]. The transmembrane domain anchors the CAR structure on the effector cell membrane [12]. Once the CAR recognizes and gets triggered by its specific antigen, the intracellular activation domain(s) of the CAR will signal, resulting in downstream processes that facilitate the killing of target cells [13, 14]. Numerous immune effector cells, like T cells, γδsT cells, natural killer (NK) cells, NKT cells, and macrophages, have been equipped with a CAR and were shown to be able to mediate anticancer responses in preclinical studies and clinical trials [4, 15, 16]. In 2017, the US Food and Drug Administration (FDA) approved the first two CAR-T treatments for CD19^+^ hematologic malignancies (axicabtagene ciloleucel and tisagenlecleucel) [1, 17].

NK cells are innate immune effector cell that can rapidly identify and kill abnormal cells, virally infected cells and tumor cells [18]. In contrast to T cells, NK cells are lymphocytes that lack antigen-specific receptors, while abundantly expressing neural cell adhesion molecule (NCAM; also known as CD56). The unique mechanism of NK to distinguish pathologic cells from normal tissue cells is determined by the combination of surface stimulatory and inhibitory receptors that recognize a wide range of ligands on target cells [19]. T cells recognize peptide in the MHC (in human called human leukocyte antigen, HLA) molecules on an antigen-presenting cell (APC) and after receiving the proper danger signals, APC activate T cells that can kill MHC class I expressing tumor cells. However, tumor cells or stressed (e.g., virus-infected) cells often exhibit reduced or no expression of MHC class I molecules and thereby become susceptible to NK cell elimination [20]. MHC class I molecules bind a suite of inhibitory killer cell immunoglobulin-like receptors (KIRs). The inhibitory KIR cluster restrains NK cell activity and thereby prevents the damage to normal “self”-cells [21]. During NK cell development, the interaction between KIRs and autologous MHC molecules provides essential signals for NK cell maturation and contributes to the acquisition of functional competency, a process termed NK cell licensing [15]. This process is part of a sophisticated mechanism known as NK cell education, in which NK cells obtain functional competence and adapt to the host where they develop [21, 22]. NK cells, unlike T cells, can also become activated after antibody binding to the CD16 surface receptor. When an antibody binds to a specific tumor antigen, this complex is bound via the Fc tail to CD16 molecules on NK cells that become activated and will eliminate the tumor cells.

An interesting property of mature NK cells for adoptive cell therapy is that they can be transplanted into a new surrounding with different MHC expression patterns without losing their function [23, 24]. In great contrast to T lymphocytes, NK cells do not induce graft-versus-host disease but rather play a regulatory role in most cases (reviewed in [25]). With the development of genetic modification technologies, NK cells have been demonstrated they can be further tailored, including the introduction of CARs and knockout of inhibitory genes [26]. Using such techniques, NK cells from patients with hematologic malignancies can swiftly kill autologous tumor cells that were previously resistant to killing by the same NK cells without a CAR [11, 27]. Preclinical studies with CAR-NK cells unraveled in vivo activity similar to that of CAR-T cells in xenograft murine models. Interestingly, the CAR-NK groups show less cytokine release and better overall survival rates [28, 29]. A first CAR-NK study in men showed a promising anti-tumor response without the development of major toxic effects, such as cytokine release storm (CRS) and graft-versus-host disease (GVHD) [30]. These encouraging results pave the way for further development of CAR-NK as an attractive modality for cancer therapy [11].

In this review, we summarize information from preclinical and clinical studies, reporting on 72 CAR-NK cell line and 35 primary CAR-NK cell investigations. We try to interpret the paradigm for CAR-NK by focusing the design of a CAR and engineering of NK cells. We describe in detail the structure of the CAR, several detection methods of the CAR on the NK cells, the ideal NK cell source for CAR-NK therapy and the techniques for ex vivo expansion of NK cells. We also discuss the approaches to deliver the CAR-containing transgene to NK cells and the methods that have been used to enhance the transduction efficiency. Finally, we will provide a comprehensive outlook on how future CAR-NK-based therapies can be used to eradicate cancer.

### A global overview of current CAR-NK cell studies

We found 72 CAR-NK cell line studies and 35 primary CAR-NK preclinical studies based on “chimerical antigen receptor and natural killer cells” from PubMed and Global data® (until March 2021). All these investigations introduce an external antigen binding motif derived from a tumor-targeting monoclonal antibody (moAb) into the cells. In Tables 1 and 2, we provide an overview of these studies using cell lines and primary NK cells, respectively. Due to space constraints, further details on the CAR design and study are made available online as interactive tables on www.carnkreview.com and are provided as Additional file 1: Table S1 and Additional file 2: Table S2.

**Table 1:** Overview of CAR-NK studies to date using cell lines

#	Reference: first author (journal, year)	Effector cell	Vehicle					Target	generation			Activation signal
			retro	lenti	mRNA	plasmid	transposon		1	2	3	
1	Uherek (Blood 2002)	NK-92	x					HER2	x			CD3ζ
2	Schirrmann (Cancer Gene Ther 2002)	YTS				x		CEA	x			CD3ζ
3	Daldrup-Link (Eur Radiol 2005)	NK-92	x	x				HER2	x			CD3ζ
4	Schirrmann (Leukemia Research 2005)	YT				x		CD33	x			CD3ζ
5	Meier (Nucl Med Biol 2008)	NK-92	x					HER2	x			CD3ζ
6	Muller (Cancer Immunol Immunother 2008)	NK-92	x					CD20	x			murine CD3ζ
7	Tavri (Mol Imaging 2009)	NK-92	x					EpCAM	x			CD3ζ
8	Boissel (Leukemia Res 2009)	NK-92			x	x		CD19	x			CD3ζ
9	Meier (Magn Reson Med 2011)	NK-92	x					EpCAM	x			CD3ζ
10	Sahm (Cancer Immunol Immunother 2012)	NK-92, NKL		2				EpCAM		x		CD28-CD3ζ
11	Boissel (Leukemia Lymphoma 2012)	NK-92		x	x			CD19, CD20	x			CD3ζ
12	Esser (J Cell Mol Med 2012)	NK-92	x					GD2	x			CD3ζ
13	Tassev (Cancer Gene Ther 2012)	NK-92-MI	x					EBNA	x			CD3ζ
14	Alkins (Cancer Res 2013)	NK-92	x					HER2	x			CD3ζ
15	Boissel (OncoImmunol 2014)	NK-92		x				CD19, CD20	x			CD3ζ
16	Jiang (Mol Oncol 2014)	NK-92-MI		3				CD138	x			CD3ζ
17	Chu (Leukemia 2014)	NK-92		2				CS1		x		CD28-CD3ζ
18	Seidel (Cancer Immunol Immunother 2015)	NK-92	x					GD2	x			CD3ζ
19	Schönfeld (Mol Ther 2015)	NK-92		2				HER2	x	x		CD28-CD3ζ/CD137-CD3ζ/CD3ζ
20	Han (Sci Reports 2015)	NK-92, NKL		2				EGFR		x		CD28-CD3ζ
21	Clémenceau (J Immunol Res 2015)	NK-92	x					Her2	x			FceRIγ
22	Liu (Oncol Reports 2015)	NK-92				x		HER2		x		CD28-CD3ζ
23	Zhao (Leukemia 2015)	NK-92-MI	x					WT-1		x		CD137-CD3ζ
24	Muller (J Immunother 2015)	YTS		2				EGFRvIII	x			DAP12
25	Alkins (Neuro-Oncol 2016)	NK-92	x					HER2	x			CD3ζ
26	Genßler et al. (OncoImmunol 2016)	NK-92		2				EGFRvIII		x		CD28-CD3ζ
27	Zhang (J Natl Cancer Inst 2016)	NK-92		2				HER2		x		CD28-CD3ζ
28	Romanski (J Cell Mol Med 2016)	NK-92	x					CD19	x			CD3ζ
29	Topfer (J Immunother 2016)	YTS		2				EGFRvIII	x			DAP12
30	Chen (Oncotarget 2016)	NK-92, PB-NK		2				EGFR		x		CD28-CD3ζ
31	Siegler (Mol Ther 2017)	NK-92	x	x				CD19, HER2		x	x	CD28-CD137-CD3ζ/CD28-CD3ζ
32	Oelsner (Cytotherapy 2017)	NK-92		2				CD19	x	x		CD28-CD3ζ/CD137-CD3ζ/CD3ζ
33	Chen (Oncotarget 2017)	NK-92		2				CD3			x	CD137-CD28-CD3ζ
34	Chen (Leukemia 2017)	NK-92		2				CD5			x	CD137-CD28-CD3ζ
35	Li (Cell Stem Cell 2018)	NK-92, iPSC					x	mesothelin	x	x	x	2B4-CD3ζ/CD28-CD137-CD3ζ/DAP10-CD3ζ/CD137-CD3ζ/2B4-DAP12-CD3ζ/2B4-DAP10-CD3ζ/CD137-2B4-CD3ζ/CD3ζ
36	Zhao (Eur J Inflamm 2018)	NK-92-MI		x				CD19, CD138		x		CD28-CD3ζ
37	Murakami (Anticancer Res 2018)	KHYG-1		x				EGFRvIII		x		CD137-CD3ζ
38	Zhang (J Immunol Res 2018)	NK-92		2				EpCAM		x		CD137-CD3ζ
39	Yu (Mol Therapy 2018)	NK-92		x				GPC3		x		CD28-CD3ζ
40	Nowakowska (Cancer Immunol Immunother 2018)	NK-92		2				HER2	x	x		CD28-CD3ζ/CD137-CD3ζ/CD3ζ
41	Tang (Am J Cancer Res 2018)	NK-92-MI		3				CD33	x	x		CD28-CD137-CD3ζ
42	Schnalzger (EMBO J 2019)	NK-92		2				HER2		x		CD28-CD3ζ
43	Ao (J Immunother 2019)	NK-92		x				AlphaFR	x	x	x	CD3ζ/CD28-CD3ζ/CD28-CD137-CD3ζ
44	Kloess (Human Gene Therapy 2019)	NK-92, PB	x					CD123			x	CD28-CD137-CD3ζ
45	Kim (Biomaterials 2019)	NK-92-MI				x		EGFR		x		CD28-CD3ζ
46	Xu (J Hematol Oncol 2019)	NK-92		3				CD5		x		2B4-CD3ζ/CD137-CD3ζ
47	Batchu (Surgery 2019)	NK-92-MI				x	x	mesothelin			x	CD28-CD137-CD3ζ
48	Kulemzin (BMC Med Genom 2019)	NK-92, YT		2				PMSA		x		CD28-CD3ζ
49	You (Am J Cancer Res. 2019)	NK-92-MI					x	CD7			x	CD28-4-1BB-CD3ζ
50	Oelsner (Int J Cancer 2019)	NK-92		2				FLT3		x		CD28-CD3ζ
51	Parlar (Eur J Immunol 2019)	NK-92, YTS		x				CD3 δγεδ, TCR	x			CD3ζ
52	Mensali (EBioMed 2019)	NK-92	x					CD3 δγεδ, TCR	x			CD3ζ
53	Ravi (Leukemia 2020)	NK-92		x				CD19	x			CD3ζ
54	Hu (Sci Report 2020)	NK-92-MI		x				Tissue factor		x		CD137-CD3ζ
55	Huang (Cancer Manag Res 2020)	NK-92		x				GPC3	x	x	x	CD3ζ/CD28-CD3ζ/2B4-CD3ζ/DNAM1-CD3ζ/DNAM1-2B4-CD3ζ
56	Mitwasi (Sci Report 2020)	NK-92-MI		x				GD2		x		CD28-CD3ζ
57	Hambach (Cells 2020)	NK-92	x					CD38			x	CD28-CD137-CD3ζ
58	Cao (Biochem Biophys Res Commun 2020)	NK-92		2				Mesothelin, CD19			x	CD28-CD137-CD3ζ
59	Li (J Cancer Res Ther 2020)	NK-92		x				Robo1		x		CD137-CD3ζ
60	Montagner (Cells 2020)	NK-92		x				PMSA		x		CD28-CD3ζ
61	Nakazawa (Anticancer Res 2020)	KHYG-1		x				EGFRvIII		x		CD137-CD3ζ
62	Robbins (eLife 2020)	NK-92	–	–	–	–	–	PDL1	x			FcεR1γ
63	Lee (J Control Release 2020)	NK-92		x				Fra, TRAIL		x		CD27-CD3ζ
64	Liu (Cytotherapy 2020)	NK-92 MI		3				CD19		x		CD137-CD3ζ
65	Yang (Front Pharmacol 2020)	NK-92 MI		2				B7H3		x		CD137-CD3ζ
66	Jamali (Front immunol 2020)	NK-92, NKL, PB		3				CD19		x		CD137-CD3ζ
67	Ahn (Biomaterials 2020)	NK-92	–	–	–	–	–	EGFR			x	CD28-DAP10-CD3ζ
68	Fabian (J Immunother Cancer 2020)	haNK-92	–	–	–	–	–	PD-L1	–	–	–	–
69	Gurney (Haematologica 2020)	KHYG-1, PB-NK	x					CD38		x		CD28-CD3ζ
70	Tseng (Nat Commun 2020)	NK-92MI, PB-NK	x	2				CD147, GPC3			x	CD28-CD137-CD3ζ
71	Eitler (Journal for ImmunoTherapy of Cancer 2021)	NK-92		2				HER2		x		CD28-CD3ζ
72	Kang (Int J Mol Sci 2021)	NK-92MI, KHYG-1		3				CD19				CD28-CD3ζ

Numbers in the "Vehicle" column refer to the generation of retro- and lentiviruses. – denotes information not provided in the study. Other abbreviations: *gen.* = CAR generation. *Lenti* lentivirus, *Retro* retrovirus, *Transp* transposon

There is an interactive and extended version of this table available online, also providing regular updates: http://www.carnkreview.com

**Table 2:** Primary cell-derived CAR-NK cells

#	Reference: first author (journal, year)	Effector cell	Vehicle					Target	generation			Activation signal
			retro	lenti	mRNA	plasmid	transposon		1	2	3	
1	Imai (Blood 2005)	PB-NK	x					CD19	x	x		DAP10/CD137-CD3ζ/CD3ζ
2	Altvater (Clin Cancer Res 2009)	PB-NK	x					CD19, GD2	x	x		CD3ζ/2B4/2B4-CD3ζ
3	Li (Cancer Gene Therapy 2010)	PB-NK			x			CD19		x		CD137-CD3ζ
4	Shimasaki (Cytotherapy 2012)	PB-NK			x			CD19		x		CD137-CD3ζ
5	Boissel (Leukemia Lymphoma 2012)	PB-NK/CB-NK		x	x			GFP, CD19, CD20	x			CD3ζ
6	De Oliveira (Hum Gene Ther 2013)	HSC		2				CD19	x	x		CD3ζ/CD28-CD3ζ
7	Chu (Cancer Immunol Res 2015)	PB-NK			x			CD20		x		CD137-CD3ζ
8	Suerth (J Mol Med 2016)	PB-NK + NKL	a/g	3				CD19			x	CD28-CD137-CD3ζ
9	Oelsner (Int J Cancer 2016)	CIK		2				CD19	x	x		CD28-CD3ζ/CD3ζ
10	Chu (OncoImmunol 2017)	PB-NK			x			CD20		x		CD137-CD3ζ
11	Kailayangiri (OncoImmunol 2017)	PB-NK	x					GD2		x	x	CD137-CD3ζ/t2B4-CD3ζ/CD137-t2B4-CD3ζ/t2B4-CD137-CD3ζ
12	Kloess (Hum Gene Ther 2017)	PB-NK	a					CD123			x	CD28-CD137-CD3ζ
13	Liu (Leukemia 2018)	CB-NK	x					CD19		x		CD28-CD3ζ
14	Oei (Cancer Immunol Res 2018)	PB-NK			x			CD19		x	x	CD28-OX40-CD3ζ/OX40-CD3ζ/CD28-CD3ζ/CD137-CD3ζ
15	Yu (Mol Ther 2018)	PB-NK		2				GPC3		x		CD28-CD3ζ
16	Li (Cell Stem Cell 2018)	iPSC/NK-92					x	Mesothelin	x	x	x	2B4-CD3ζ/CD28-CD137-CD3ζ/DAP10-CD3ζ/CD137-CD3ζ/2B4-DAP12-CD3ζ/2B4-DAP10-CD3ζ/CD137-2B4-CD3ζ/CD3ζ
17	Kloess (Hum Gene Ther 2019)	PB-NK/NK-92	a					CD123			x	CD28-CD137-CD3ζ
18	Oberschmidt (Hum Gene Ther Meth 2019)	PB-NK	a					CD123			x	CD28-CD137-CD3ζ
19	Colamartino (Front Immunol 2019)	PB-NK		2				CD19, CD22			x	CD28-CD137-CD3ζ
20	Ingegnere (Front Immunol 2019)	PB-NK/NK-92				x		CD19	x	x		CD3ζ/CD137-CD3ζ
21	Herrera (Sci Rep 2019)	CB-NK/PB-NK		3				CD19		x		CD137-CD3ζ
22	Bari (Front Immunol 2019)	PB-NK		3				CD19		x		CD137-CD3ζ
23	Wang (Blood Advance 2020)	PB-NK	x					BCMA, CD123	x			CD3ζ
24	Liu (NEJM 2020)	CB-NK	x					CD19		x		CD28-CD3ζ
25	Muller (Front Immunol 2020)	PB-NK	a	3				CD19		x		CD28-CD3ζ
26	Quintarelli (Leukemia 2020)	PB-NK	x					CD19		x		CD137-CD3ζ
27	Ueda (Cancer Sci 2020)	iPSC		x				GPC3			x	CD28-CD137-CD3ζ
28	Liu (Cell Proliferation 2020)	PB-NK		2				EGFR		x		CD137-CD3ζ
29	Gang (Blood 2020)	PB-NK		3				CD19		x		CD137-CD3ζ
30	Yang (Mol Ther Methods Clin Dev 2020)	PB-NK	x					CD19		x		CD137-CD3ζ
31	Wilk (Blood Advances 2020)	PB-NK			x			CD19	–	–		
32	Daher (Blood 2020)	CB-NK	x					CD19		x		CD28-CD3ζ
33	Jamali (Front Immunol 2020)	PB-NK/NK-92/NKL		3				CD19		x		CD137-CD3ζ
34	Gurney (Haematologica 2020)	PB-NK			x			CD38		x		CD28-CD3ζ
35	Tseng (Nat Commun 2020)	NK-92MI, PB-NK	x	x				CD147, GPC3			x	CD28-CD137-CD3ζ

Numbers in the "Vehicle" column refer to the lentivirus generation. For retroviruses: a = alpha, g = gamma. – denotes information not provided in the study. Other abbreviations: *gen.* CAR generation, *Lenti* lentivirus, *Retro* retrovirus, *Transp* transposon, *PB-NK* peripheral blood-derived NK cells, *CB-NK* cord blood-derived NK cells, *CIK* Cytokine-induced killer cells

There is an interactive and extended version of this table available online, also providing regular updates: http://www.carnkreview.com

**Fig. 1:**
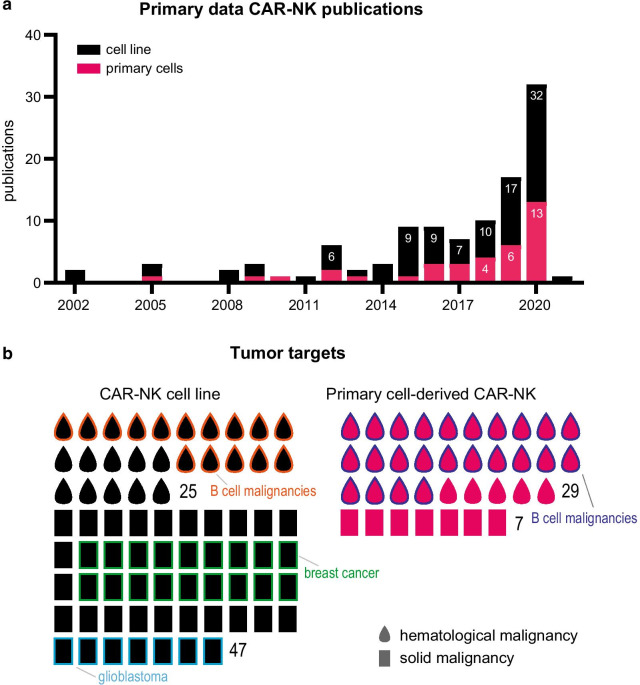
CAR-NK cells: growing interest and diversity of tumor targets. **a** Bar graph showing the number of manuscripts reporting experimental data on human CAR-NK cells until December 2020. Stacked bars show the number of publications per year with CAR-NK cells derived from cell lines (black) and primary NK cells (pink). **b** Tumor targets for cell line-derived (black) and primary NK cell-derived (pink) CAR-NK cells. Each symbol represents one study with experimental data, with blood drops for hematological malignancies and rectangles for solid malignancies. Numbers per tumor type are shown, with annotated boxes highlighting the most important tumor targets

The number of CAR-NK preclinical studies is increasing year by year, describing both CAR-NK cell lines and primary CAR-NK cells (Fig. 1a). In this review, we will zoom in on the various CAR elements and the techniques required to generate CAR-NK cells for clinical applications. At first glance, the overview of the current CAR-NK studies already shows interesting trends. In CAR-NK cell line studies, Her2 (expressed on a subset of breast cancer cells) is the most used target for solid tumors, while the CD19 antigen (B cell malignancies) is the most popular in hematological cancers (Fig. 1b). Of the primary NK cell studies, 65% use primary CAR-NK cells investigating B cell malignancies with CD19 as the most favorite target (Fig. 1c). Interestingly, the number of solid tumor CAR-NK cell lines studies are over 2 times higher than of hematological malignancies. In the following chapters, we will discuss the various components of that make up a successful CAR-NK cell.

## CAR structure design

**Fig. 2:**
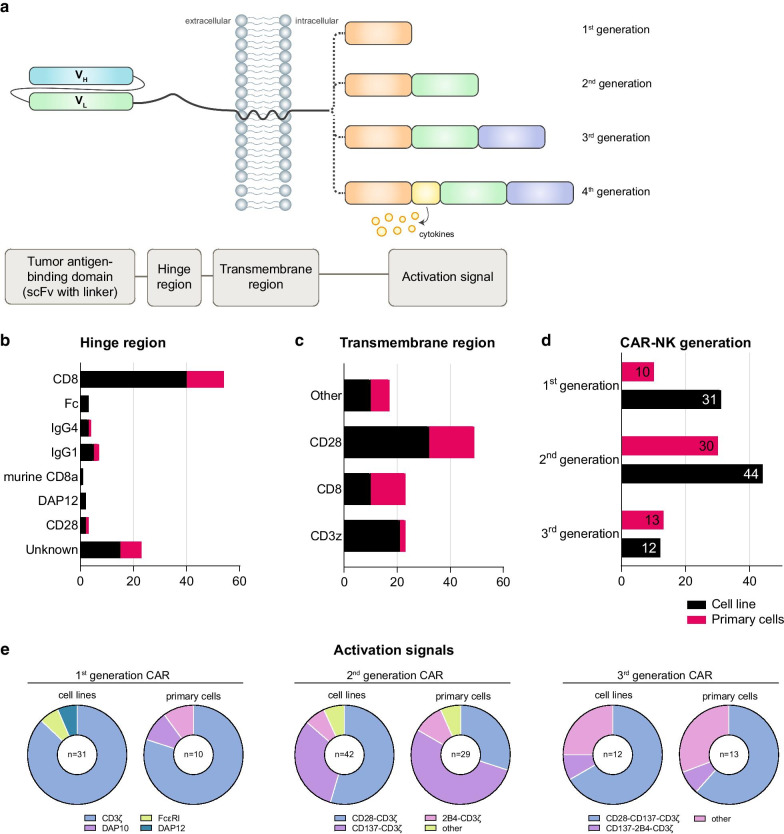
CAR-NK cell design. **a** Schematic overview of the building blocks for CAR-NK cells. From left to right: the extracellular single-chain variable fragment (scFv) consists linker-bound heavy and light chain and it determines the specificity of the CAR. The hinge region connects the tumor antigen-binding domain to the transmembrane region, ensures CAR stability and provides the flexibility for easy access to the target protein. The transmembrane region joins the extracellular and intracellular CAR domains, docks the CAR to the cell membrane and transduces activation signals to the intracellular activation signals. The number of intracellular activation domains determines the CAR generation. Various combinations of activating domains are used to mount a strong anti-tumor response. One example of a “fourth-generation” CAR is shown, co-expressing stimulating cytokines. Other examples of next-generation CAR constructs are discussed in Table 3. **b** and **c** Analysis of the most frequently used hinge (**b**) and transmembrane (**c**) regions for CAR-NK construction. Only studies reporting on the hinge region were included for this analysis. **d** Quantification of experimental studies with the indicated CAR-NK generations for cell line-derived (black) and primary NK cell-derived (pink) CAR-NK cells. **e** Pie charts showing the activation signals used for cell line-derived and primary cell CAR-NK cells, separated by CAR generation. Only the most frequently used combinations are shown

Functional CAR molecules expressed on NK cells consist of three parts: the ectodomain, transmembrane region and the endodomain (Fig. 2a). The ectodomain is made up of a signal peptide, the single-chain fragment variant (scFv) with a linker between the heavy chain and light chain and the hinge region that connects this structure to the transmembrane region. This latter region docks the CAR molecule to the cell membrane. It also connects intracellularly to the endodomain that encompasses the activating signals. The combination of these building blocks, together with regulatory elements that are situated outside the open reading frame (e.g., promotor), will determine the efficacy of the CAR. Successful CAR design is achieved by a combination of careful in silico design and functional testing. In this section, we will discuss the various building blocks of a CAR in detail and will summarize the current knowledge of successful combinations of these components. This information, often combined with software that assists in the analysis of expression levels, protein folding and spatial confirmation resulting from a certain DNA and protein sequence [31], will aid in successful CAR design. Before discussing CAR design, we will focus on the vector that carries the transgene and facilitates the insertion into the effector cell.

### Vector backbone and promoter

The vector backbone contains all elements needed for expression of the CAR such as the promoter, the polyadenine signal and transcriptional regulation fragments.

As promoters initiate CAR transcription, the choice for a certain promoter has a direct influence on expression levels of the transgene. Furthermore, Allan et al. have recently reported that the size of the promoter influences the viral transduction efficiency and subsequent GFP expression in a negative correlation [32]. In their study, the shorter EFS, PGK, SV40 and CMV promotors are good promoters that can maintain the GFP expression for 17 days [32]. CAR genes are usually introduced using viral plasmids with an exogenous promoter. Currently, there is only one comparison of different promotors on CAR expression and function in NK cell lines published, while no comparative data are available for primary NK cells [33]. From the single report on CAR-NK cells, no definitive statements can be made about the best promotor for CAR-NK cells [33]. In primary T cells, various comparisons of the effect of promoters on CAR expression levels were performed. One study shows that the EF1α promotor induced higher CAR expression levels compared to other commonly used promotors, such as CMV, UbiC and PGK [34]. Another study showed that the MSCV outperformed both EF1α and PGK in terms of expression levels and CAR expression stability [35]; however, the promotor efficacy may be influenced by the scFv sequence itself [36] (see section Single-chain fragment variant (scFv)). Given these and other studies [37] that report different optimal promoters, it is important to determine the optimal promoter for a given construct (that may also contain additional elements that influence expression levels, such as reporters) and viral vector. The latter is important, since the choice of a promoter also influences the virus titer. For instance, one study reports that the CMV promoter sequence yielded higher virus titers compared to RSV promoter sequences when located within the SIN 5′ LTR lentiviral vector [38]. Besides baseline CAR expression in the immune cell, the dynamic ubiquitination, down-regulation and re-expression kinetics following antigen exposure will have large impact on the efficacy of CAR cells [39].

Compared with the retroviral LTR promoter-based CAR that integrates “randomly,” including in potential oncogenic sites, it may be desirable to make use of site directed insertion into a target cell specific promotor area. An interesting example is the use of the endogenous TRAC promotor where the CAR gene was deliberately integrated into the *TCR alpha* chain gene locus [40]. This concept was already introduced as a way to avoid GVHD in T cell-based adoptive cell therapy [41]. Eyquem and colleagues took this a step further by demonstrating that CAR-transduced lymphocytes integrated in the *TRAC* locus persist longer in murine tumor models [40]. In addition, more effector cells infiltrated into the tumor and exhibited a less exhausted cell phenotype [40]. TRAC-CAR-T outperformed both conventional retroviral transduced CAR T cells (RV-CAR) as well as T cells with the CAR inserted in the β2M locus (β2M-CAR) in an in vivo mouse model, based on tumor eradication and expression of exhaustion markers, including PD1, TIM3 and LAG3 [40]. In addition, TRAC-CAR increased CAR expression levels after antigen stimulation, while CARs driven by an exogenous promotor showed important downregulation. Although this CAR expression under an endogenous NK cell-specific promoter has not been reported, GFP or HA epitopes have successfully been expressed in primary NK cells by CRISPR/Cas9-mediated knock-in into the *CLTA* [42], *AAVS1* [43], *CD96*, *ACTB* and *RAB11A* gene loci [26]. Thus, the use of endogenous promotors should also be further explored in the CAR-NK cell field.

Current reports on CAR-NK cells show a wide variety of promoters being used for driving CAR expression, both for cell line-derived and primary NK cells. In both primary CAR-NK and CAR-NK cell lines, viral promoters (CMV, MPSV, MMLV, SFFV, etc.) are more frequently used to generate CAR-NK than constitutively active promoters, such as EF1α, CMV and PGK (80% and 78% for primary and cell line CAR-NK, respectively; Additional file 1: Table S1 and Additional file 2: Table S2).

### Signal peptide for CAR-NK

The CAR sequence starts with a short signal peptide (SP). These are short peptides located at the N terminus of proteins, carrying information for protein post-translational assembly in the endoplasmic reticulum (ER) and Golgi organelle and expression on the membrane [44]. The final CAR protein on NK cells will be a type I transmembrane protein that anchors on the cell membrane. CAR, like many other proteins, is also secreted by immune cells via the co-translational translocation pathway [45]. In eukaryotic cells, SP located at the N terminus of nascent proteins are identified by the signal recognition particle (SRP) while the protein is still translating in the ribosome. After the nascent protein crosses the ER membrane, the signal peptide is cleaved off by a signal peptide peptidase (SPP), and then protein assembly and folding will commence [46].

There is enormous heterogeneity in signal peptides, which directly translates into different levels of protein secretion efficiency. For both CAR-NK and CAR-T cells, we did not find comparative studies that determined the optimal signal peptide for CAR expression and function. At present, CD8a SP is the most used peptide sequence for primary NK (16%, with data missing in 71% of the studies; Additional file 2: Table S2) and immunoglobulin heavy or light chain SP for NK cell lines (29%) (Additional file 1: Table S1). While the SP sequence is likely to influence CAR expression levels, there are currently no data to support claims about the optimal SP for CAR-NK generation.

### Single-chain fragment variant (scFv)

The single-chain fragment variant (scFv), a fusion protein of the variable regions of the heavy and light chain of an antibody, is the tumor antigen-binding domain of the CAR. Different scFv could bind to different epitopes of the same protein, and therefore, this domain will determine both the specificity and the function of the CAR-NK cell [47]. For instance, targeting Mucin 1 (MUC1) which is a glycosylated, abundant protein, many antibodies have been described and various scFvs have been created in CAR constructs [48, 49]. Diverse scFvs lead to various anti-tumor capacities but also distinct side effects, such as binding to normal tissue. Therefore, the target of a CAR needs to be selected carefully to be specific for the tumor-associated antigen, while at the same time not recognizing self-antigens that could induce severe side effects [1].

However, the numerous efforts undertaken to find a suitable target on solid tumors illustrate how difficult the process of scFv selection can be. This is largely due to the expression of most so-called tumor-associated antigens on both tumors and—often at lower levels—in healthy tissue. There already have been reports of the potential damaging effects of CAR-engineered T cells, such as the 4D5-scFv anti-ERBB2 [50] and G250-scFv anti-CAIX [51].

As the scFv is not a naturally occurring form of an antibody, it is always synthesized, and therefore the order of the heavy and light chain is artificially determined. Thus far, most groups prefer a VH-VL orientation over VL-VH for CAR-NK design (Additional file 1: Table S1 and Additional file 2: Table S2). Fujiwara et al. demonstrated that the order of H and L chain did not influence the expression level of anti-KDR CAR expression on T cells [36]. In addition, scFv performance in terms of antigen specificity and association constant in a CAR is sometimes found to be lower compared to its originating antibody, mostly due to the changed connectivity of the VL and VH domains. This may also have implications for the dynamics and relative orientation of the two chains. Computational-assisted design of a scFv may help in the development of functional scFv by analyzing the amino acid sequence of the CDR region of the VH and VL, and predicting the interaction of the scFv with its target [52]. Interestingly, VH and VL chains from different antibodies against the same epitope can also be mixed, sometimes leading to increased CAR affinity [53].

The importance of scFv design was underlined by a study that shows distinct scFvs targeting the same antigen may yield very different results in terms of CAR expression [36]. In this study, CAR-T cells that expressed functional CARs were detected, but also CAR-T cells with no or very low levels of antigen binding. Here, changes in the CDR region accounted for the observed differences, while there were no effects due to a specific VH/VL order [36]. Another example was published very recently and showed that from the 8 differently derived scFvs specific for CD19 when lentiviral transduced as CAR into the NK cell line KHYG-1, only two of them were efficiently expressed on the cell surface and showed improved cytotoxicity towards CD19 + tumor cell lines [54]. However, whether this was due to the specific sequence of the scFv as the CDR3 regions do vary, or a different spacer and transmembrane domain remains to be elucidated [54].

In addition, immune cells can be equipped with multiple scFv, thus broadening the antigen recognition capacity of a CAR effector cell. Here, there are various options: CARs can be transduced with vectors with bi-cistronic elements, inducing expression of two CAR constructs that may or may not have the same intracellular structure. In addition, two scFvs can be fused together in one construct, generating a tandem scFv or “single stalk” CAR. While these techniques have been employed for the generation of CAR-T cells [55], we are not aware of such attempts for generating CAR-NK cells.

Most current clinical CAR T cell trials have utilized scFv derived from murine antibodies, thereby increasing the risk for an anti-mouse IgG cell host-versus-graft disease, resulting in toxicity or shortened persistence of CAR-bearing cells [6]. This issue may be attenuated by humanizing murine scFv or by deriving scFv from fully human antibodies. In previous studies that employed murine scFv-based anti-CD19-CAR or anti-CD5-CAR, the NK-92 cells exhibited strong anti-tumor activity to ALL malignant cells both ex vivo and in a mouse model [56, 57]. An anti-GD2-CAR with humanized scFv showed similar expression levels as its murine counterpart, while increasing cytokine release and T cell proliferation [58]. Unfortunately, owing to the chimeric character of these CAR receptors, even humanized scFv constructs still may induce a host anti-idiotype immune responses [59]. Fortunately, in the limited number of CAR-NK clinical trials so far, no major side effects related to the anti-CAR immune responses were found [30]. Above all, from these studies, both murine and humanized svFv derived CAR-NK exhibit robust cytotoxicity against antigen-expressing tumor cells both in vitro and in vivo.

### Linkers

The linker between the heavy and light chain contributes to the conformation of the scFv and therefore partly determines how well a CAR recognizes the target epitope. Linkers that are too short induce the formation of multimers, because they prevent association of the VH and VL domains. Too long domains, on the other hand, may induce proteolysis or weak association between the VH and VL domains [6]. For CAR-NK cells, multimers of the pentapeptide GGGGS (glycine-serine) are most widely used, often as a 15-mer (G_4_S)_3_. Another linker designed to enhance proteolytic stability is the Whitlow “218” linker: GSTGSGSKPGSGEGSTKG. In one study, this linker enhanced scFv affinity, possibly altering CAR-NK cell function as well [60]. Fujiwara et al. compared G_4_S and 218 linkers, but, in their anti-KDR-CAR-T, there were no expressional and functional differences between (G_4_S)_5_, (G_4_S)_3_ and 218 linkers [36].

Currently, there are insufficient data available to draw conclusions about the best linker for CAR. We found that 18 studies used G_4_S linkers, whereas 2 publications applied 218 linkers among the CAR-NK cell lines (Additional file 1: Table S1). While most primary CAR-NK reports did not provide specifics on the linker, all 4 studies that provided these details used G_4_S linkers to generate CAR-NK (Additional file 1: Table S2).

### Hinge region (CD8α, Ig CH2CH3)

The hinge region, also referred to as a spacer, is the extracellular structural region of the CAR that connects the scFv units to the transmembrane domain. These spacers generally maintain the stability required for robust CAR expression and activity in effector cells. The hinge region also supplies flexibility to allow easy access to the targeted antigen. The majority of CAR-NK constructs use derivatives of CD8α or CD28 extracellular domains or immunoglobulin G (IgG)-based hinges (Fig. 2b). Accumulating evidence shows that the type and length of the spacer importantly influence the functional activity of the CAR [6]. As no specific data are available for CAR-NK cells, this part of information is all derived from the CAR-T field, and it remains to be shown if this can be directly translated to CAR-NK design.

In a direct comparison between the CD28 and CD8α hinge domains, it was found that CD28 hinge domains are more likely to promote dimerization of CAR molecules [61]. As a result, the activation stimulus derived from CD28 hinge-bearing CARs is stronger [61]. While this may be beneficial, this could also induce cytokine release syndrome, one of the most severe side effects of CAR-based therapy. Indeed, in a clinical study aiming to improve the safety of anti-CD19-CAR-T therapy, it was found that an optimized CD8α hinge region, together with other modifications, importantly improved the safety profile of the therapy [31].

Immunoglobulin G-based hinges are also widely used in CAR constructs. A major advantage of an IgG-based hinge region is the flexibility of the construct that is typically composed of IgG_1_ or IgG_4_ and the CH2/CH3 domains of IgG Fc. The use of the latter domains is optional and thus allows for modulation of the length of the spacer. While a wide range of spacer lengths will accommodate antigen recognition, studies have found that shorter spacers were associated with increased cytokine production, CAR-T cell proliferation and better persistence and antitumor effects in vivo [62].

For CAR-NK cells, we found that most studies employ a CD8α hinge region, both in primary NK cell (16/35) and CAR-NK cell lines (41/72) (Fig. 2b). Other spacers, such as CD28, IgG Fc domains and DAP12 were also used in CAR-NK.

### Transmembrane domain (CD3, CD8, CD28, NKG2D, 2B4)

The transmembrane (TM) domain connects the ectodomain of the CAR to the intracellular activation signaling domains and docks the receptor to the NK cell membrane. The most commonly used TM parts of CAR-NK have been adapted from CD3ζ, CD8 and CD28, but others (e.g., NKG2D, 2B4, DNAM1) have been explored as well (Fig. 2c). The choice of the TM domain was shown to influence the functionality of the CAR construct in the extent of cell activation. The TM domain of CD28, CD16, NKp44, NKp46, NKG2D, DNAM-1 and 2B4 have been used to screen for CAR function using the NK-92 cell line. Interestingly, the TM from molecules typically expressed on NK cells, like DNAM-1, 2B4 and NKG2D leads to more CD107a degranulation and higher cytotoxicity. Thus, the specific source of the TM will determine the activity of CAR-NK [28].

One important aspect of the TM domain is that the optimal TM region should follow the natural orientation (N- to C-terminal order) of the protein from the transmembrane protein on the T cells or NK cells [63]. In this paper, Guo et al. addressed the use of the NK-native molecule NKG2D as a TM region and activator signal for NK-92 cells [63]. NKG2D is a powerful activator of NK cells and thus constitutes a suitable backbone for NK-focused CAR design. However, natural NKG2D has the transmembrane region in the C- to N-terminal order with a short cytoplasmic tail. Interestingly, the use of a signal peptide to forcibly reverse the NKG2D TM region to the N- to C-terminal order, showed in combination with 2B4 and DAP10 the strongest cytotoxic effects in iPSC-derived CAR-NK cells [28]. But whether this is really due to the reversed order alone cannot be concluded as the Kaufmann group focused on identifying the best combination of intracellular signaling domains.

These CAR-NK studies underline that it is important to consider the TM region in its function as a linker to the intracellular activation signal, as the type of TM may influence signal transduction and CAR dimerization. In CAR-T cells, CD28-derived TM domain was shown to promote activation-induced cell death (AICD) and cytokine production, whereas CARs with a CD8α-derived TM assisted in CAR dimerization with endogenous TCRs, leading to first activation signal of T cells [61]. At this moment, CD8α and CD28-dervied TM are most popular in primary CAR-NK cells, while CD28 was the preferred TM region for CAR-NK cell lines (Fig. 2c).

### Activation signal for CAR-NK

CARs are often identified by their respective generation. The number of intracellular activating signals determines this “generation”: First-generation CARs have one activating signal, second-generation CARs have two, and so forth (Fig. 2a, d).

The activating domains are responsible for the activation of the NK cell upon recognition of the target antigen. In contrast to T lymphocytes, NK cells use a variety of different, non-rearranging receptors for activation. This also includes a large variety of cytokine receptors that are important regulators of development, maturation and activation of NK cells [64]. Most of these receptors share common adapter molecules and signaling pathways. For instance, NKp30 and CD16 both signal via CD3ζ [65]. The cytokines IL-2, IL-7, IL-12, IL-15, IL-18, IL-21, IL-27, and IFN-α/β signal via the highly conserved JAK/STAT pathway [64]. Thus, even though the first activation signal in T cells is provided through a clonally rearranged antigen receptor, many of the downstream signaling pathways are shared between T and NK cells. Therefore, some insights from the CAR-T field also apply to CAR-NK cells. At the same time, it is important to realize that CAR-based signaling differs significantly from canonical activation pathways. For instance, in normal T lymphocytes, activation depends on the stabilization of multimolecular complexes (TCR-peptide-MHC with co-receptors) and recruitment of co-stimulatory molecules, whereas these signals are provided "in line" in CAR-equipped cells. This has direct consequences for the temporal availability of the various activating molecules involved. We have only just begun to grasp the importance of these differences between canonical signaling and CAR signaling. The findings from the CAR-T field (summarized by Lindner and colleagues [66]) will again serve as an important starting point for studying these differences in CAR-NK cells as well.

First-generation CAR-NK cells, like CAR-T cells, only contain the CD3ζ signal. The second- and third-generation CAR-NK bear one and two additional co-stimulatory signals, respectively. The costimulatory molecules are usually derived from the CD28 family (including CD28 and ICOS), the tumor necrosis factor receptor (TNFR) family of genes (including 4-1BB, OX40 and CD27) or signaling lymphocytic activation molecule (SLAM)-related receptor family (comprising 2B4) [67]. To these activating domains, safety switches can be added to quickly eliminate the infused CAR-containing cells in the case of adverse reactions occur, such as the CRS [30, 68] (this aspect will be further discussed in the Prospective and outlook section). In addition to this example, other modifications to the effector cells have been developed that improve persistence, enhance tumor activity, prevent antigen escape, allow for control of CAR expression or combinations of these (Table 3). An in-depth discussion of these next-generation CAR constructs is beyond the scope of this review, also because almost all development took place in the CAR-T field. The only published CAR-NK clinical trial so far employed a second-generation CAR-NK construct that was enhanced with IL-15 expression and inducible Caspase 9 [30]. CAR-T cells that include cytokines enhancing persistence and anti-tumor activity are popularly referred to as fourth-generation CARs (Fig. 2a) or TRUCKs: T cells redirected for antigen‐unrestricted cytokine‐initiated killing. Two recent examples of CAR-NK enhancements show that the CAR-NK field follows these developments, adapting T cell-specific signals to NK cells where needed. A first example of the translation of a CAR-T cell enhancements to CAR-NK cells was recently provided by Wang and colleagues [69]. They developed CAR-NK cells with a protein switch that enhances NK cell proliferation and survival, while at the same time coupling CAR expression with ectopic IL-15 expression and an inducible suicide gene [69]. In recently published other study, the expression of a CAR/IL-15 construct (“fourth-generation CAR”) is coupled with CRISPR/Cas9-mediated knockout of *CIS*, a negative regulator of IL-15 signaling [70]. This interesting strategy boosted CAR-NK cell function in vitro and in xenograft models, at least in part through increased aerobic glycolysis. This double enhancement of IL-15 signaling is likely to be beneficial in the tumor microenvironment that is often limited in IL-15 levels [70]. Modification of cytokine signaling in CAR-NK cells may thus provide further possibilities for CAR-NK cell improvement. For instance, cytokines like IL-12, IL-15, IL-18 are responsible for the induction of memory-like NK activity following CMV infections [71, 72] or even anticancer responses [73]. Future studies will provide more insight in the other enhancement strategies work best for CAR-NK cells, helping them to overcome challenges posed by the tumor microenvironment (also see section Challenges).

**Table 3:** Next-generation CAR-NK cells

Enhancement strategy	Target	Aim	References
Cytokine co-expression	IL-12	Improve persistence	Koneru [203]
		Improve anti-tumor activity	Pegram [204]
	IL-15		Liu [30]
			Krenciute [205]
	IL-18		Avanzi [206]
			Hu [207]
Cytokine/JAK/STAT co-expression	IL-2Rβ + STAT3/5	Improve persistence	Kagoya [208]
		Improve anti-tumor activity	
Cytokine receptor co-expression	IL-7Rα	Improve persistence	Shum [209]
		Improve anti-tumor activity	
Chemokine co-expression	CCR2, CCR2b, CCR4, CCR7, CXCR2, CXCR4	Promote trafficking into tumor microenvironment	Brown [210]
			Craddock [211]
			Moon [212]
			Rapp [213]
			Di Stasi [214]
			Carlsten [215]
			Kershaw [216]
			Hillerdal [217]
Dual CAR (two antigens required for activation)	BCMA + CS1	Enhanced safety and efficacy	Chen [218]
Split CAR (separation of co-stimulatory domains)	PSMA + PSCA	Achieve tumor-specificity in the absence of a truly tumor-restricted antigen	Kloss [219]
Multi-antigen targeting (bi-specific CAR)	HER2 + IL13Rα2	Improve anti-tumor activity	Hegde [220]
	HER2 + IL13Rα2 + EphA2	Prevent antigen escape	Bielamowicz [221]
Universal CAR	CD16 (Fc receptor)	Precise control of CAR reactivity based on Ab half-life	Caratelli [222]
	Antibody tag	Re-use of approved antibodies, requiring only one CAR construct	Tamada [223]
			Feldmann [224]
	FITC		Tamada [223]
Inhibitory CAR Controllable CAR expression systems	Healthy tissue antigen, e.g., CD19 (with inhibitory domain, e.g., PD-1, CTLA-4)	Improve specificity, better discrimination healthy and tumor tissue	Fedorov [225]
	Syn/Notch	Controlled CAR expression	Morsut [226]
	Inducible co-stimulation	Inducible CAR activation	Mata [227]
Knockout of checkpoint inhibitor	PD-1	Improve persistence	Rupp [228]
		Improve anti-tumor activity	Ren [229]
			Cherkassy [194]
			Daher [70]

Examples of enhancements to CAR constructs, mostly derived from the CAR-T field

Most current CAR configurations depend on the CD3ζ chain signaling domain, yielding 3 immunoreceptor tyrosine-based activation motifs (ITAMs) per CAR. In turn, these ITAMs will recruit and activate the Syk or ZAP70 tyrosine kinases, or induce PI3-kinase signaling [22]. As CAR-NK cells are usually also designed around the CD3ζ domain, and again specific studies regarding activation signals in CAR-NK cells are currently lacking, we must rely on findings from the CAR-T field regarding the molecular makeup of downstream activation.

Strong activation signals are important to induce a potent anti-tumor response, but can also lead to quick exhaustion of the effector cells. Therefore, the combination of co-stimulatory domains can be used to calibrate the desired immune cell response. Compared to 4-1BB-based CARs, CD28-based CARs exhibit an effector profile that is faster and provokes larger-magnitude changes in lymphocyte-specific protein tyrosine kinase (Lck) phosphorylation in the signaling pathway [74]. In this way, CD28-triggered signals induce higher levels of interferon-γ (IFN-γ), granzyme B, tumor necrosis factor α (TNF-α) [74]. However, it is known from CD28-based CAR-T cells that this strong co-stimulatory signal also causes activation-induced cell death (AICD) and that the weaker CD3ζ ITAM signals lead to better CAR-T cell function and longer persistence [75, 76]. On the other hand, 4-1BB-CD3ζ signals preferentially induce memory-associated genes and sustained antitumor activity [74]. The reason may be that antigen-independent tonic signaling through CD28 domains increases T cell exhaustion while the presence of the 4-1BB domain ameliorate this [77]. The implications of tonic signaling through CAR structures were covered in an excellent review by Ajina and Maher [78].

Moreover, activation signals may have an impact on the metabolism in immune cells. CD28-CAR cells are predominantly dependent on glycolytic metabolism, while 4-1BB-containing CAR cells exhibited superior persistence as a result from increased oxidative metabolism [79]. It was recently found that calibration of the CD3 ITAM region importantly changes the functional phenotype of CAR-T: Introduction of mutations in different parts of the CD3ζ ITAM motifs leads to a naïve-like T cell phenotype, with high proliferative capacity and longer persistence [76]. On the other hand, NK cells may be induced into cells with a memory-like phenotype after activation with IL-12, IL-15 and IL-18 [80]. When an anti-CD19 CAR was introduced in NK cells activated a memory phenotype, these cells showed enhanced in vitro and in vivo anti-tumor activity [80].

Another study shows that there is a lot of room for improvement of the activation signals in CAR cells. In CAR-equipped T cells, proximal signaling downstream of the antigen receptor was significantly reduced compared to normal T cells, due to inefficient recruitment of ZAP-70, resulting in important deficiencies in downstream signaling [81]. Thus, the choice of activation signal will have great impact on the functional and persistence of CAR-bearing cells. As these studies were conducted in T cells, there is an important need for structured analysis of the optimal combination of activation domains for NK cells.

We found 72 publications describing CAR-NK cell lines using 90 constructs and 35 studies with 43 different constructs using primary CAR-NK cells (Fig. 2e). Both in CAR-NK cell line and primary CAR-NK cell studies, CD3ζ is almost universally used as the main activation domain of which about half carry one additional domain, usually adding 4-1BB or CD28. As for third-generation constructs, combination of CD28/4-1BB/CD3ζ is most often used. These constructs thus provide a means for NK cells to directly receive co-stimulation signals once the CAR binds the tumor antigens. Given the disparities in both antigen sensitivity and number of ITAMs, it is plausible that the smaller number of ITAMs per CAR receptor could result in a comparatively longer persistence of CAR-T cells [76].

To our knowledge, the only direct comparison of different activation signals in CAR-NK was performed by Li et al.[28]. In that study, among 9 different constructs, a 2B4-CD3ζ-based CAR-NK construct showed the most robust CD107a degranulation and antigen specific cytotoxicity. Moreover, a point mutation of Arg-to-Ala in the transmembrane or Tyr-to-Phe in the ITAM/ITSM of NKG2D-2B4-CD3ζ construct sections reduces phosphate recruitment of downstream activation molecules like pSyk, pPLC-γ2 and pERK1/2. This will decrease degranulation, cytokine release and antigen-specific lysis capacities of the CAR-NK cells. This indicates that the transmembrane and intracellular domains are also crucial parts that determine CAR-NK cell functionality.

In addition, the combination CD28-CD3ζ was also shown to provide strong activation signals and is able to support CAR-NK cell survival for one year in vivo in the first published clinical trial with CAR-NK cells [30]. In general, the CD3ζ domain is most often used (Fig. 2e), but it still remains to be determined which is the best combination of domains, in which order and in which situation.

### Detection tags (GFP, cMyc-tag, FLAG, LNGFR)

Introduction of molecular tags and fluorescent proteins together with CAR genes allows for easy approaches to enrich, quantify and trace the CAR-NK. For instance, various groups use c-Myc or Flag tags directly situated before or after the scFv, which are then expressed together with the CAR on NK cells [82]. Fluorescent proteins are also commonly used in CAR-NK plasmids using bi-cistronic elements, and allow researchers to conveniently trace the expression level of CAR in NK cells [83]. The disadvantage of this latter system is that the detection of the tag does not always reflect the CAR expression (our own unpublished data). A second issue in this case is that fluorescent proteins and other big epitopes are foreign proteins to the human body and could thus induce an immune response directed to this exogenous epitope. Therefore, these tags are generally used in the laboratory setting and are removed when clinical studies are started.

### Synthetic biology: codon optimization and scFv humanization

Codon optimization is a technique used to alter the use of nucleotides without changing the amino acid sequence. For CAR-NK design, many researchers rely on the use of DNA synthesis for some of the building blocks for their plasmids. Because of technical limitations during synthesis, it may be necessary to change the DNA sequence to allow for successful production. Indeed, this codon optimization was mentioned in 8 out of 72 CAR-NK cell line and 5 out of 33 primary CAR-NK reports. It is important to realize that the use of a certain codon set may improve CAR-NK expression in mammalian cells, but may have negative effects on virus production [84]. In one direct comparison of CAR-T cells with and without codon optimization, no differences were found in CAR surface expression, tumor eradication and cytokine production [85].

The starting point for the development of new CARs often is murine antibodies recognizing a tumor-associated antigen. However, murine antibodies cannot directly be used for clinical use, as the non-human regions of the mouse antibody are often immunogenic. Therefore, these regions can be replaced with corresponding human sequences in a process called “humanization.” Indeed, this technique is also successfully applied for the development of CAR scFv without negative effects on CAR-NK killing capacity [58, 86].

## Transfection or transduction vehicle for CAR expression

**Fig. 3:**
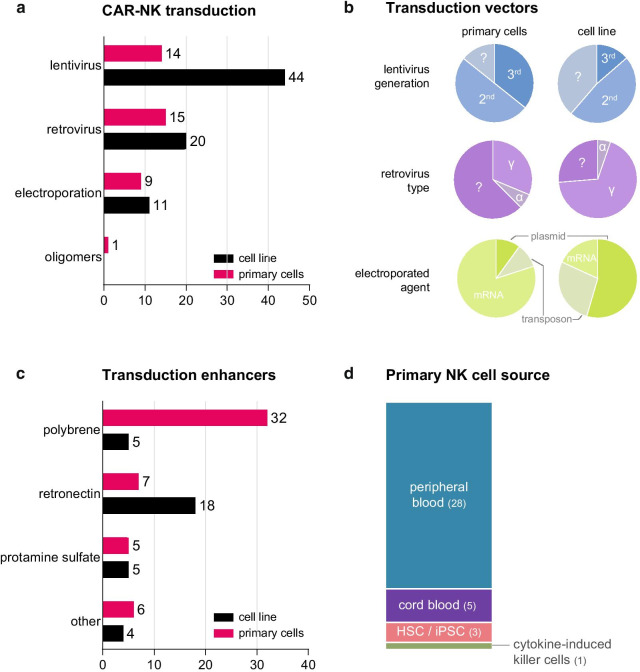
Optimization strategies for CAR-NK cell generation. **a** Different vectors can be used to transduce NK cell lines and primary NK cells with CAR constructs. Quantification of the vectors used for CAR-NK generation. **b** Details of the various vectors used for CAR-NK generation. In blue, lentivirus generation, with third-generation viruses considered the safest option with minimal risk of in vivo recombination with other lentiviruses. In purple, the genus of retrovirus (alpha or gamma, or unknown). In green, the electroporated vector. **c** Various compounds have been identified that can be used to enhance virus-mediated transduction. For studies that employ virus-based strategies for CAR-NK generation, the methods used for enhancing virus transduction were quantified. **d** Primary cells for generation of CAR-NK cells are derived from different sources. Quantification of the four sources for primary NK cells for CAR-NK generation

With the progress in gene modification technologies, numerous approaches have been applied to generate CAR-NK. The two main methods are viral transduction (using lenti- or retroviruses), or transfection with either naked plasmid DNA, transposase DNA-mediated integration or mRNA by electroporation [14] (Table 4). A total of 64 preclinical studies describing CAR-NK cell lines using viral transduction and 11 studies applying electroporation or nanoparticle-mediated transfection have been published to date. In primary CAR-NK cell studies, 29 described viral transduction and 9 publications conducted electroporation as method of choice (Additional file 1: Table S1, Additional file 2: Table S2 and Fig. 3e). Monoclonal or polyclonal NK cell lines can be generated using FACS or (Clini-) MACS, but this creates a more complex good manufacturing practice (GMP)-compliant production process. An important difference between the various technologies is the duration of stable CAR expression. For longer expression (multiple weeks) in primary cells, viral transduction is usually employed, while mRNA electroporation results in transient expression lasting for about one week. CAR expression levels in primary NK cells strongly vary (20–70%) and are thus sometimes low, but most studies still report highly specific and effective killing of target-positive tumor cells.

**Table 4:** Comparison of virus- and non-virus mediated CAR delivery to NK cells

Method	Advantages	Disadvantages	GMP compliance
Lentivirus	High efficiency	Potential genotoxicity owing to LTR sequences	Third generation is considered safe enough for clinical use
	Favorable safety profile	Difficult to obtain high titer LV	
	Transduction of resting NK		
Retrovirus	Long history in clinical trials	Risk of insertional oncogenesis	Compatible; successfully used to generate CAR-NK for clinical trials
	Relatively easy to produce	Transgene capacity is lower than LV	
		Requires actively dividing cells	
mRNA electroporation	Cheaper than viral production	Transient expression	Compatible
	No integration risks		
	High transduction rates		
Transposon	Transgene capacity higher than for viruses	Risk of insertion into genome	Compatible
	Cheap, easy to produce		
CRISPR/Cas9	Precise gene modification	Complicated to design the HDR template	Not yet applied in CAR-NK clinical trials
	Ability to use endogenous promoter of choice	Efficiency is hard to control	

*LTR*: Long terminal repeat, *LV*: lentivirus, *HDR:* homology-directed repair

### Lentiviruses

Lentiviruses have been widely used in the gene therapy space for decades, as they are able to transduce cycling and non-cycling cells with high transduction efficiency. They have been successfully applied in curing people from diseases including HIV and cancer [87]. Lentiviruses have the advantages that they integrate into the host genome, a feature that can lead to permanent expression of the transgene, and low intrinsic immunogenicity [88]. To date, there are 14 reports on primary CAR-NK cells and 44 papers on CAR-NK cell lines that have successfully used a lentivirus as vehicle (Fig. 3a). Among preclinical studies, 21 studies used a second-generation virus and 6 studies used a third-generation lentivirus to generate a CAR-expressing NK cell line (17 unknown) (Fig. 3b). In the primary CAR-NK cell studies, 5 studies used third-generation lentivirus while 7 reports describe second-generation lentiviral vectors (2 unknown) (Fig. 3b). The third-generation lentiviral vectors are being considered safer than the second-generation due to *gag/pol* and *rev* viral genes that are located on separate plasmids, thus making wild-type recombinant virus generation even more unlikely [89]. In order to improve the fraction of CAR-expressing cells after transduction, CAR-NK cells can be enriched using fluorescence-activated cell sorting (FACS) or through antibiotic selection. Most studies applied a third-generation lentivirus under GMP conditions for clinical application and often showed a transduction efficiency lower than 10% on NK cells [90], explained by the low binding capacity of the VSV-G envelope to the LDL-R, which is the receptor facilitating entry of the virus. Changing the virus pseudotype (e.g., to BaEV) was suggested to enhance the viral transduction efficiency as it targets other receptors expressed at higher levels in NK cells [91, 92]. Furthermore, stimulation with cytokines or certain compounds can facilitate higher transduction rates in NK cells [92–94] (discussed further in Chapter 5). All combined, these results have demonstrated that lentiviruses are a powerful vehicle to manufacture CAR-NK cells for clinical disease.

### Retroviruses

Retroviruses have been used as gene therapy vectors for decades [95]. Retrovirus-based vectors require NK cells to be actively dividing to obtain integration of the vector into the genome [96]. There are 19 studies using CAR-NK cell lines and 16 reports using primary NK cells published that employ retroviruses (Fig. 3a). One recent Phase I clinical trial targeted CD19^+^ non-Hodgkin's lymphoma and chronic lymphocytic leukemia by infusion of retrovirus-transduced anti-CD19 CAR cord blood NK cells. In this study, 73% of patients responded with 7 out 8 patients attaining complete remission. Moreover, responses were fast and seen within 30 days after CAR-NK infusion at all dose levels. After one-year follow-up, expanded CAR-NK cells could still be detected by qPCR [30]. After infusion, CAR-NK DNA copy numbers remained stable for up to one year in peripheral blood and measured between 10^2^ and 10^4^ copies of the CAR-NK vectors per μg genomic DNA. These results show for the first time that retrovirally transduced CAR-NK cells can persist in vivo for a long time.

Different genera of retroviruses have been used to generate CAR-NK cells (Fig. 3b). Alpha retroviruses carrying the RD114 envelope are superior in transduction efficiency of primary NK cells compared to gamma retrovirus and lentivirus [97]. Recently, Muller et al. showed that RD114 alpha-retroviruses result in 3 times higher CAR-NK transduction efficiencies (around 45%) than VSV-G lentiviruses on day 3 in primary NK cells (about 15%) [91]. However, from day 7 onwards, retroviruses and lentiviruses showed equal performance [91]. Retrovirally transduced CAR-NK cells can be expanded with sustained CAR expression for at least 2 weeks. Although long and stable CAR expression in NK cell can be achieved using different retroviruses, safety of the retrovirus system is still a concern, especially when compared to safer lentiviruses.

### Electroporation of mRNA

Electroporation of CAR encoding mRNA is a swift and efficient but transient approach to generate CAR-NK cells. Some companies already developed equipment to facilitate electroporation in a closed system compatible with CAR-NK cell generation, which is especially interesting for GMP-compliant production starting from primary NK cells. There are 11 studies in CAR-NK cell lines and 10 primary CAR-NK cells that rely on electroporation (Fig. 3a). The electroporated cargo mainly consists of CAR-encoding mRNA or plasmids (Fig. 3b). With the current technological advances and the use of high-purity CAR mRNA instead of cDNA in a plasmid, transfection efficiencies in NK cells have increased dramatically, achieving up to 95% with minimal negative effects on cell viability [98, 99]. Generally speaking, mRNA transfection efficiencies are much higher in expanded or activated NK cells (more than 60%) than in freshly isolated NK cells (about 40%) [100]. As mRNA synthesis is compatible with GMP regulations, and electroporation can be done in a clean room, it is thus feasible to generate GMP-compliant CAR-NK via mRNA electroporation. However, the main disadvantage of this method is the narrow, transient window of CAR expression: After electroporation, the CAR-NK cells should be transfused back into patients within 7 days.

### Sleeping beauty transposon

Transposon-based systems can introduce CAR transgenes with higher efficiency and at predefined locations, which is an important advantage over conventional methods that do not possess an integrating element. Transposons are mainly introduced into NK cells by electroporation followed by integration into the host genome through transposase enzymes [101, 102]. Two studies applied the transposon system to generate CAR-NK cells: One used NK-92-MI cells [101], the other study described transposon transfection into iPSC cells followed by differentiation into NK cells [28] (Fig. 3a). Both studies used the 4D-Nucleofector electroporator to introduce the transposase encoding plasmids into cell nucleus (Fig. 3b). After enrichment, anti-mesothelin-CARs were stably expressed on iPSC-derived NK cells and were functional in a murine model of ovarian cancer [28]. Although there are many studies using the transposon system to generate primary CAR-T cells [53, 103], primary NK cells are much more difficult to transduce using transposons. We expect that with further advances in transposon and transfection methods, generation of CAR-NK cells using transposon will become a more viable approach.

### CRISPR/Cas9-mediated strategies

CRISPR/Cas9 has recently emerged as a powerful technique for genetic modification. This technology relies on the introduction of Cas9 protein in conjunction with guide RNA into the NK cells. Initially, this technique was used in primary NK cells to disrupt the *CD38* gene, aiming to prevent fratricide of NK cells when they were used in combination with daratumumab (anti-CD38), as CD38 is expressed both on NK cells and multiple myeloma and AML cells [104]. More recently, CRISPR/Cas9 has also been applied to introduce new genes [105]. Here, in parallel to CRISPR/Cas9, a homologous donor DNA template is introduced in the same cell via transfection [106]. This DNA template replaces the targeted gene, thus allowing for the introduction of genes that promote the anti-tumor effects. This technique was first successfully applied in primary T cells: CRISPR/Cas9 was used to target the TCR alpha gene, relying on homology-directed repair (HDR) to knock-in a CD19-CAR cassette. The expression of the CD19 CAR, now under the control of the endogenous TCR alpha promoter, manifested much stronger expression that also persisted longer compared to lentiviral vectors using a viral promoter [40, 107]. Following this paradigm, one can apply this concept to generate more potent and persistent CAR-NK cells. In several studies that employ HDR templates, knock-in efficiencies of more than 75% were obtained using K562-mIL-21-expanded NK cells (see also the section on Cytokines and K562-mIL-21/4-1BBL feeder cells) [43]. However, in fresh NK cells, the Cas9 knock-in efficiency is only 3–16% [42]. When the CAR gene is delivered via viral vectors or using transposon-based random insertion, expression levels are variable. CAR expression driven by an endogenous promotor following CRISPR/Cas9-specific locus knock-in strategies could result in long CAR-T persistence in vivo, with a memory-like phenotype and less expression of exhaustion markers [40]. Thus, the CRISPR/Cas9 strategy is a versatile technique that should be further explored for the generation of CAR-NK cells. It can be used to precisely delete, repair or introduce genes in a specific locus, hence holding promise to generate powerful anti-tumor NK cells [43].

## CAR-expression detection methods

Following transduction of the chimeric antigen receptor, various techniques are available to assess CAR expression levels on the cell surface and to determine the efficacy of the developed CAR-NK cells. The most employed experimental modalities include flow cytometry, (fluorescence) microscopy and analysis of gene expression levels. In addition, flow cytometry and gene copy number analysis are used to monitor CAR-NK cells in patients. Here, we discuss the advantages and disadvantages of the various approaches.

### Flow cytometry

Flow cytometry is widely used to detect CAR expression, since it is a fast and reliable detection method that provides quantitative data on protein expression at the single cell level. This technique relies on the use of fluorescently labeled antibodies that recognize parts of the CAR complex on the membrane. In addition, flow cytometry can be used to detect fluorescent tags or tags that were specifically added to the transduction cassette to facilitate expression recognition as is introduced in section Detection tags (GFP, cMyc-tag, FLAG, LNGFR).

Labeled antibodies or their derivates, such as antigen-binding fragments (Fab), can be used to detect components of the CAR complex on the cell membrane. Commonly, these antibodies target the antigen-recognizing scFv part of the CAR. In addition, the CAR ligand with a fragment crystallizable region (Fc) tail is used in conjunction with Fc-recognizing labeled antibodies. While these methods have the advantage that they are very specific for the detection of a specific CAR, this is also the main drawback of this approach: Some antibodies are generally not commercially available [108].

To overcome this challenge, universal detection methods have also been developed. For instance, protein L, a bacterial-derived product that recognizes the variable part of the light chain, binds to most immunoglobulin classes, including scFv that are used in CAR development [109]. Protein L has successfully been used to detect the expression of murine and human CAR on peripheral blood cells, including NK cells [110].

Another strategy comprises the incorporation of tags specifically included to facilitate the detection of the CAR. For this, molecular tags such as polyhistidine-tag (“His-Tag”), FLAG, or c-Myc tags have been incorporated into the CAR construct to facilitate easy detection using commercially available monoclonal antibodies [111]. Even though these tags are small and considered lowly immunogenic [112], it is undesirable to include non-essential and foreign sequences in products designed for clinical use.

Indeed, in the experimental setting other detection methods can also be used to help in CAR detection. For instance, fluorescent tags (e.g., GFP) or non-native receptor proteins, such as LNGFR [113], can be added to the CAR construct. While these methods simplify CAR detection, it is important to realize that fluorescent proteins or additional receptors are included in the CAR construct using bi-cistronic elements, or their expression is driven by additional promotors. Thus, the expression of the tags may not reflect CAR expression and may only be a marker of successful transduction.

### Quantitative PCR

Quantitative PCR is a well-established technique for quantification of CAR transgene expression levels. As the technique requires disruption of individual cells to isolate genomic material, this technique does not provide direct information about CAR expression at the single cell level. However, surrogate markers that are expressed at relatively stable levels (housekeeping genes) can be used to estimate the number of cells and to follow copy numbers over time. Another limitation of PCR-based techniques is that they do not provide information on viable effector cells, nor do they take into account that not all transferred cells will be *bone fide* CAR-expressing effector cells.

Given these limitations, qRT-PCR is mostly used for follow-up studies in clinical trials in which flow cytometry cannot be used due to the low frequency of circulating CAR-positive effector cells. As an example, in a recently published phase I/II trial using CAR-NK to target CD19-expressing malignancies, gene copy number analysis was used to measure in vivo expansion of CAR-NK [30]. The viral vector transgene copy numbers were normalized to the amount of genomic DNA. Using this technique, the authors were able to show expansion of CAR-NK cells as early as 3 days after infusion and demonstrated that CAR-NK cells persisted in patients for at least 12 months.

Novel techniques, such as droplet digital PCR (ddPCR), allow for absolute quantification of single molecules using an advanced microfluidics system [114, 115]. The main advantage of this technique is that it eliminated the need for a standard curve, and therefore allows for reproducible analysis of very low copy numbers. This is likely to be important of the CAR-NK field, as the number of CAR-NK cells in a patient is relatively low, especially at later time points. However, at this moment, ddPCR has important disadvantages: Sample handling is much more complicated, time-consuming, expensive and relies on small-size chips or cartridges, disqualifying the technique for large numbers of samples.

### Western blot

Western blots that employ antibodies recognizing an antibody, as for flow cytometry, can also be used to detect CAR expression in a cell population [111]. Immunoblots do not provide information at the single cell level, are time-consuming, and therefore are not routinely used to confirm CAR incorporation. However, they can provide valuable information about the size of the complex in which CAR is incorporated and are thus often used in CAR optimization studies. For instance, several studies have shown that CARs need to form dimers to be responsive and that they need to engage in a complex with signaling domains to be effective [116].

### Confocal microscopy

Fluorescent and electron microscopy can be used to visualize CAR molecules on the surface of effector cells. Similar to flow cytometry and western blot, this technique relies on the use of antibodies that can be CAR-specific, or universal antibodies that recognize common CAR components. Indeed, fluorescent and electron microscopy have been used to detect CAR molecules on the surface of transduced NK cells [117]. Given that microscopic analysis is low-throughput, these techniques may not routinely be used to confirm CAR expression, but can be valuable tools to visualize the correct incorporation of the CAR in the plasma membrane and to determine the number of CAR molecules per cell.

### Molecular imaging

In addition to follow-up by flow cytometry and determination of gene copy numbers, various molecular imaging strategies are currently under investigation for experimental and clinical use. Following transduction, cells are loaded with tags that can be detected using optical, nuclear or magnetic resonance techniques [118]. While fluorescent and bioluminescence techniques are only viable for use with laboratory animals, positron or photon-emission tomography (PET/SPECT) and magnetic resonance imaging can also be used in humans. These techniques can be used to gain insight in the NK cell biology at the cellular and molecular level, as they allow for quantitative tracking of (CAR-) NK cell biology in vivo [118]. However, most of these techniques are still in the very early phases, requiring complicated and expensive labeling procedures and long acquisition times.

## Approaches for enhancing NK cell virus-mediated transduction

Lentiviruses and retroviruses are the most commonly used systems to induce stable expression of chimeric antigen receptors in NK cells. Encouraging results have been shown for the transduction of NK cell lines and primary NK cells, including their use in clinical trials [89, 119]. However, attaining high viral transduction efficiency while maintaining good cell viability remains a challenge, especially for primary NK cells. Various strategies have been developed to enhance viral transduction of NK cells (Fig. 3c).

These approaches often target the entry of a vector into NK cells, either via changing electric charges of cells or by targeting the viral envelope or the receptor on the target cell [120, 121]. Therefore, changes to the envelope on the viral surface and up-regulation of receptors on the target cell are the main approaches to boost transduction [89, 120].

### Enhancing NK cell transduction via upregulation of LDLR using statins

Most lentiviruses used for gene transductions are pseudotyped with a VSV-G envelope glycoprotein. The main receptors for this envelope protein are the low-density lipoprotein receptor (LDLR) and phosphatidylserine [122]. NK cell lines and primary NK cells express low levels of LDLR. Interestingly, statins that are used in the clinic to lower blood cholesterol levels were shown to induce LDLR on B and T lymphocytes [123]. Similarly, we found that statins also induced LDLR levels in NK-92 and primary NK cells [93]. Consequently, transduction efficiency increased after treatment with statins. However, statins have also been reported to suppress the cytotoxic capacity of NK cells, which can be completely reversed by geranylgeranyl-pyrophosphate (GGPP). Not all statins are potent boosters of viral transduction: Rosuvastatin in combination with GGPP most potently improved viral transduction without affecting the cytotoxic properties of the NK cells [93].

### Other small molecular compounds to enhance NK cell transduction

In addition to statins, other compounds have been tested to enhance the viral transduction of NK cells. Negative charges existing on both the viral envelope glycoproteins and the target cell receptors can be detrimental for transduction [124]. Therefore, cationic polymers, such as protamine sulfate, polybrene [125] and dextran [126], can be used during transduction to positively charge the cell surface [124] (Fig. 3c). Using high-throughput analyses, other compounds that enhance lentiviral transduction have also been identified. Phorbol 12-myristate 13-acetate (PMA) [127], rapamycin [128], Cyclosporin A [129], P338 poloxamer [130], prostaglandin E2 (PGE2) [131], vectofusin-1 [132] and RetroNectin [92] have all been reported to promote lentiviral transduction on hematopoietic stem cell (HSC), T cells or B cells. However, in our analysis on the effects of these compounds on NK cell transduction, we found that only statins consistently promote viral transduction without major negative effects on cell viability [93].

Next to compounds that promote viral transduction, substances that inhibit antiviral responses in NK cells can also be used to enhance transduction. Indeed, BX795, an inhibitor of the TBK1/IKKε complex that controls antiviral responses in NK cells, was demonstrated to promote lentiviral gene transduction efficiency by 3.8-fold [133].

### Alternative lentiviral pseudotypes for NK cell transduction

VSV-G has long been used as the major glycoprotein for pseudotyping lentiviruses because of its broad tropism, facilitating the transduction of a wide range of cell types [134]. VSV-G lentiviruses have also been used to generate genetically modified NK cells for decades, and make stable NK cells line [135, 136]. However, as alluded to above, the low expression levels of the VSV-G receptor LDLR on fresh human NK cell hinder efficient and easy lentiviral transduction of NK cells [93, 94]. As an alternative to boosting LDLR expression levels, other glycoproteins can be used to pseudotype the viral particle [121].

Colamartino et al. showed that BaEV lentiviruses outperform VSVG-, MV- and RD114-pseudotyped viruses in human NK cells, even using activated NK cells [92]. Their study supports the idea that the abundance of the lentivirus receptor on the target NK cell may correlate with the efficiency of the integration of the gene of interest. The receptors for BaEV are ASCT1 and ASCT2, which are highly expressed on activated NK cells, both at the mRNA and protein level [92, 94]. Therefore, BaEV lentiviruses are potentially superior to VSV-G lentiviruses for gene therapy in NK cells [92].

RD114 pseudotyped retroviruses have also been used to generate human NK cells expressing an anti-CD19-CAR [137]. Suerth et al. demonstrated that efficient modification of human NK cells by using an alpha-retroviral vector with a RD114/TR envelope [97]. VSV-G-pseudotyped lentiviral particles normally outperform VSV-G pseudotyped alpha-retroviral and gamma-retroviral particles. However, when alpha-retroviral particles were pseudotyped with RD114/TR, this resulted in superior transduction performance. These results show that the compatibility of virus particles and the surface glycoprotein will ultimately determine the efficiency of viral transduction [97]. With these concepts, further studies towards modifying the component of the viral glycoprotein specific for NK cells may improve the LVs transduction efficiency [121].

### Cytokines and K562-mIL-21/4-1BBL feeder cells

The cytokines and feeder cell lines that are used to expand NK (see section NK cell expansion) cells may also have positive effects on transduction with retro- and lentiviruses. Previous studies demonstrated that IL-2 induced upregulation of the viral receptor LDLR in NK cells, resulting in enhanced transduction, proliferation and cytotoxicity [138]. The addition of soluble IL-2, IL-12 and IL-21 to the NK cell medium has also been reported to enhance VSV-G lentiviral transduction, with a possible synergistic effect of additional PHA [133, 135]. Other reports have indicated that culturing primary human NK cells with K562 feeder cells with membrane-bound IL-21 and 4-1BBL also boosts lentiviral and retroviral transduction of NK cells [92, 139]. Thus, these methods are already used and could be easily implemented to further enhance viral transduction.

### Microfluidic mechanic devices

The titer of the virus is a crucial parameter of the viral transduction. Although with the advancement of viral vectors and transfection technologies, it remains difficult to fully standardize the method to obtain a high enough and consistent titer to perform a successful transduction. Even though this system has not been tested for NK cell transduction, the microfluidic platform systems developed by Luni et al. [140] and Tran et al. [141] may develop into a new versatile tool to improve the transduction efficiency while working at low viral titers in a sequential manner.

## Cell sources for CAR-NK generation

NK cell-based immunotherapy requires relatively large numbers of effector cells. The life span and proliferative capacity of NK cells is limited, and extensive ex vivo handling of NK cells might influence their cytotoxic capacity. Multiple sources have been used to isolate and generate sufficient NK cells for CAR-NK applications (Fig. 3d and Table 5). In this section, we will discuss the advantages and disadvantages of these NK cell sources for the development of CAR-NK cells.

**Table 5:** NK cell sources

NK cell source	Advantages	Disadvantages
Peripheral blood NK cell	Commonly used source	Low transduction efficiency
	Mature NK cells	Expansion induces telomere shortening and reduced cytotoxicity
	Easily expanded ex vivo	Heterogeneity
Cord blood-derived NK	High proliferative capacity	Low expression of natural cytotoxicity receptors
	Safety and persistence demonstrated in CAR-NK trials	Heterogeneity
Stem cell-derived NK cells	Off-the-shelf product	Potentially immunogenic
	Homogeneity	Potential of malignant transformation
	Less cytokine release but still high killing capacity	
NK-92 and other NK cell lines	Off-the-shelf product	Tumor-derived cells, requires irradiation
	Stable expression of CAR on the surface	Irradiated CAR-NK-92 cells are not sustained in vivo
	Irradiated CAR-NK-92 cell already used in clinical trials	Not capable of ADCC (without additional modifications)

### Peripheral blood NK cells (PB-NK)

NK cells can be isolated relatively easily from the patient itself (autologous PB-NK) or from healthy donors (allogeneic PB-NK), and therefore most (31/33) preclinical CAR-NK studies use PB-NK. The majority of these studies use allogeneic NK cells from healthy donors (Fig. 3d).

For cancer immunotherapy, autologous NK cells are typically not very effective, as they are functionally silenced when they encounter self-MHC antigens. Furthermore, the function of patient-derived NK cells is often compromised by the underlying disease or previous treatment. Therefore, allogeneic PB-NK are often preferred for immunotherapy purposes, even though this requires careful depletion of the T lymphocytes from the product, as these latter cells could induce GVHD [142].

Since NK cell numbers in peripheral blood are relatively low, NK cells are routinely expanded after isolation. Even though the number of CAR-NK cells required for a meaningful response has not been established, previous NK cell-based therapies typically infused 10^5^–10^8^ cells/kg, thus requiring as many as 10^10^ NK cells for a patient weighing 100 kg. Therefore, various expansion protocols have been developed, which will be discussed further in this review (section NK cell expansion).

The main advantage of PB-NK cell sources for cancer immunotherapy is that the cells are already mature, and do not need to undergo a lengthy differentiation protocol, as for stem cell-derived sources. On the other hand, the transduction efficiency of PB-NK is relatively low (discussed in sections “Transfection or transduction vehicle for CAR expression” and “Approaches for enhancing NK cell virus-mediated transduction”), and prolonged culture often leads to telomerase shortening and reduced cytotoxicity due to exhaustion, even though these problems can be largely circumvented by using the correct combination of stimulatory molecules [143–145].

### Cord blood-derived NK cells (CB-NK)

A possible limitation of PB-NK cells is the dependency on the availability of healthy donors at the time the NK cell expansion process needs to be started. In a clinical setting where the new diagnosis of cancer warranting CAR-NK treatment cannot be planned, this may pose a problem in logistics and planning. Therefore, alternatives starting from frozen material, becoming off-the-shelf products, are an attractive alternative. However, it has been reported that freezing of mature PB-NK cells importantly decreases their viability and cytotoxic capacity [146, 147]. In general, large numbers of NK cells can be obtained from umbilical cord blood samples, due to the high proliferative capacity of these cells. For instance, only 10% of one cord blood unit is required to generate an almost pure pool of more than 10^9^ NK cells in two weeks [117], which is usually sufficient for one treatment cycle. Another advantage of CB-NK cells is that the haplotype of the sample can be determined at the time of collection, allowing the generation of a cell bank from which HLA-mismatched NK cells can be selected on demand.

There are also some concerns about the cytotoxic capabilities of CB-NK cells. The expression of natural cytotoxicity receptors is lower in CB-NK cells that were expanded in an artificial antigen-presenting cell system compared to IL-2-expanded cells. However, the expression levels of KIRs, NKG2A, CD94 and NKG2C were similar and no differences in cytotoxicity in killing multiple myeloma cells were observed [148].

Although only a minority of the preclinical CAR-NK studies uses cord blood as a cell source (Additional file 2: Table S2), the potency of CB-NK was recently confirmed in the first publication of a clinical trial using CAR-NK cells [30]. Here, CB-NK cells were expanded on K562-mbIL21 and 4-1BB ligand feeder cells and endowed with a CD19-directed CAR, ectopic IL-15 production and an inducible suicide gene. Remarkably, qPCR showed that these CB-CAR-NK cells persisted more than 270 days in vivo [30]. It is yet to be established if this finding correlates with long-lasting clinical responses as well.

### Stem cell-derived NK cells: differentiation of NK cells from hESC and iPSC

Disadvantages of PB-NK and CB-NK as cell sources for CAR-NK development include the natural heterogeneity between donors, resulting in variance in performance of the final NK cell product. For that reason, stem cell-derived NK cell expansion is an attractive alternative to develop a standardized, off-the-shelf therapeutic product.

Indeed, clinical-scale NK cell production starting from human embryonic stem cells (hESC) or induced pluripotent stem cells (iPSC) has been demonstrated [149–151]. The use of commonly available hESC/iPSC cell lines is often preferred over the use of stem cells from bone marrow biopsies, G-CSF mobilization or human embryos [144, 145]. Next to ethical and practical issues attached to the use of these sources, the outcome of NK cell production starting from primary stem cells is less predictable and often less efficient (reviewed in [152]). The production of NK cells starting from hESC or iPSCs takes 3–5 weeks and is therefore significantly longer than NK cell expansion starting from peripheral blood- or cord blood-derived NK cells.

When considering the use of hESC-derived NK cells, it is important to note that NK cells derived from the H9 hESC cell line showed limited allogeneic immune response, while they showed a more mature cytotoxic phenotype compared to CB-NK [150]. While we are not aware of any preclinical or clinical studies using hESC to generate CAR-NK cells, it is probably technically feasible.

On the other hand, iPSC-derived NK cells that were already shown to be functional against tumors [151, 153] have recently been used to generate CAR-NK cells. After optimization of the activation domains for the use in iPSC-derived NK cells, mesothelin-targeting iPSC-CAR-NK cells were shown to be as potent as CAR-T cells [28]. Interestingly, tumor-bearing mice treated with CAR-iPSC-NK showed less pathogenic organ damage and lower IFN-γ and IL-6 levels than CAR-T-treated in a murine xenograft model.

There are still challenges ahead before iPSC-NK cells can be safely used for the generation of CAR-NK for clinical application. Firstly, CAR-iPSC-NK cells ceased proliferating in vivo after the exogenous administration of cytokines was stopped in the murine model. Systemic administration of cytokines in a clinical setting is highly undesirable, since it is not only costly, but also potentially dangerous. Secondly, iPSC-derived cells always bear the potential of malignant transformation. Even though there were no signs of transformation in the present studies, this point may not be neglected during long-term observations. Lastly, iPSC-derived cells are potentially immunogenic, which may lead to the destruction of the effector cells, or even to adverse immune reactions, such as cytokine release storms.

### NK cell lines: NK-92

The aforementioned NK cell sources have one major disadvantage in common: Obtaining large numbers of NK cells is relatively cumbersome and time-consuming. In general, cell lines circumvent most of these problems, as they are easy to maintain and expand. Therefore, it is not surprising that 72 experimental studies have reported the use of NK cell lines for the development of CAR-NK cells. More than 80% of these studies use the lymphoma-derived NK-92 cell line [154]. Out of the six NK cell lines available, NK-92 has high anti-tumor activity and is capable of direct cytotoxicity. When NK-92 cells are transfected with CD16, this cell line can also trigger antibody-dependent cell-mediated cytotoxicity (ADCC) [155].

For CAR-NK cell development, NK-92 cells have another main advantage: the cell line also can be easily genetically modified using non-viral methods, including electroporation [156]. So far, NK-92 cells have been tested in preclinical CAR-NK studies targeting AML (directed to CD33), lymphoma (CD19), myeloma (CS1), prostate cancer (EpCAM), breast cancer (Her2), neuroblastoma (GD2), glioblastoma (EGFR) and ovarian cancer (mesothelin). Details on these trials can be found in Table 1 and Additional file 1: Table S1.

One of the major disadvantages of the NK-92 cell line is that the cells are tumor-derived and are aneuploid [154]. Therefore, they need to be irradiated before infusion, preventing in vivo proliferation and limiting their lifespan. In phase I trials, NK-92 administration was shown to be safe with only minor reactions [157, 158]. The number of head-to-head comparisons of CAR-equipped primary NK cells versus NK-92 cells is limited. In a study using a CD123-targeting CAR, primary NK cells were shown to be less effective than NK-92 cells in the eradication of AML cells in vitro [159]. This was possibly due to lower surface expression levels of the CAR in primary NK cells. However, it is difficult to draw a concrete conclusion whether the irradiated CAR-NK-92 cells is outperforming the primary CAR-NK cells on overall survival of patients, since the NK-92 cells are replication-deficient. Therefore, their persistence needs further investigation.

With only one CAR-NK clinical trial completed, it is impossible to draw conclusions on the usability of NK cell lines for CAR-NK therapy. Results from the CAR-T field indicate that long-term persistence is important for achieving and attaining complete remission [160]. While NK-92-CAR cells will not sustain in vivo, it is relatively easy to perform multiple infusions of this cell product. Further studies will be necessary to compare how NK-92-CAR can be used in the clinic.

## NK cell expansion

Allogeneic NK cells have been proven safe and efficient to treat patients with AML and ALL [23, 24]. However, to achieve the number for clinical therapy a large-scale GMP expansion of NK cells ex vivo is necessary as the numbers present in peripheral blood of a donor is not sufficient. Several methodologies have been described to facilitate a successful NK cell expansion (Table 6). Expansion can be achieved culturing the cells in various cytokines mixtures (IL-2, IL-12, IL-15, IL-18 and/or IL-21], K562 cell-based cytokine transfected cells (e.g., with IL-15 or IL-21 in combination with 4-1BBL), or autologous PBMC stimulated cells. With the development of automatic and closed expansion equipment, like Miltenyi’s Prodigy, Lonza’s Wave and G-Rex static bioreactors, it has become feasible to expand NK cells according to GMP requirements.

**Table 6:** Approaches for NK cell expansion

Approach	Advantages	Disadvantages
Cytokine mixture	Feeder free	Relatively long procedure
	Easy	May induce NK cell exhaustion
K562 or other feeder cell-based expansion	Rapid expansion	Risks from the feeder cells (contamination, tumorigenesis)
	Elongation of telomeres (only with IL21)	
Autologous PBMC feeder cells	Safe	Need for autologous cells
	Well-known	Expansion limited compared to other techniques
Super agonist-based expansion	Feeder free	Technology is complicated
	In vivo expansion	

However, the time it takes to reach clinically relevant numbers may be very long as exemplified by Lapteva et al. who showed that their cytokine mixture needed expansion of NK cells during 4–10 weeks [161]. Although this is biologically relevant, for large-scale production the time it takes is too long and thus not commercially and practically interesting.

It is also important to note that NK cells are generally easier to genetically modify after they have been activated by cytokines. While this is an important advantage, it also implies the need for an increased amount of the transduction agent. Obviously, a balance needs to be sought here, which is also clear from the fact that most studies report that viral transduction of NK cells was conducted after 7 days of activation and expansion.

The advancement of NK cell expansion technologies also facilitates CAR-NK cell generation. Co-culture of NK cells with the leukemia cell line K562, often transduced with membrane bound cytokines, recently gained attention as a relatively easy way to obtain large numbers of NK cells. After 7 days of expansion with K562-mIL-21/4-1BB-L feeder cells, the transduction efficiency of expanded NK cells can be above 80% compared to below 20% on freshly isolated NK cells [92]. In total, at this moment there are 13 studies published that used the K562 cells as feeder cells for CAR-NK cells to expand, including 12 studies using K562-mIL-15 feeder cells, 3 groups used K562-mIL-21 cells and 1 publication used the parental K562 cell. NK cells expanded with mbIL15 showed reduced telomere lengths, while those propagated with mbIL21 showed more elongated telomere lengths than freshly isolated peripheral blood NK cells [143]. This suggests that mbIL-21 is an attractive option to expand cells over many generations to reach clinically relevant cell numbers. Clinically relevant expansion levels have been reached in 2 weeks [162] (and our own unpublished data).

## Prospective and outlook

### Progression in CAR-NK clinical trials

The number of clinical trials in the CAR-NK cell space is limited (Table 7). Although in recent years, the first 2 papers showing evidence of clinical successes have been published [30, 163], many of the investigations are still in early planning or recruiting into clinical trials. Most of these trials are company-driven, with the majority coming from China (Table 7). In these trials, various diseases are targeted, with an anti-CD19 CAR (B cell malignancies) on NK cells initially being compared the same CAR on T cells. Furthermore, different tumor antigens, like HER2, Mucin-1, EpCAM or PMSA, are targeted on a variety of solid and hematological tumors (Table 7). In the clinical studies currently conducted, CAR-NK cells are derived from PBMC, the NK-92 cell line or a newly generated uniform cell line NK101. In contrast, Rezvani and colleagues have used expanded umbilical cord NK cells, selected on a KIR mismatch between donor and recipient and promising results were reported [30].

**Table 7:** Registered CAR-NK clinical trials

Origin	NCT number	Target	Extra feature	Source of NK cells	Indication	Trial Phase (Ph)	Trial status	Sponsor and country
Industry	GDCT0392921	EGFR	Daratumumab	FcRγ-deficient NK (PB-derived)	Glioblastoma	Ph 0	Planned	Indapta Therapeutics, USA
	GDCT0392823	NKG2D	None	Allogeneic, off-the-shelve	AML/MDS/liver cancer	Ph 0	Planned	NKarta, USA
	Not available	EGFR	None	iPSC-derived NK	Glioblastoma	Ph 0	Planned	Cytova Therapeutics, USA
	NCT04050709	Her2	PD-L1	NK-92 cell line	Locally Advanced Solid Tumor	Ph I	Ongoing, recruiting	NantKwest Inc, USA
	NCT04052061	CD19	None	NK-92 cell line	B Cell Lymphoma	Ph I	Ongoing	NantKwest Inc, USA
	NCT03692663	PSMA	Cyclophosphamide	NK-92 cell line	Prostate Cancer	Ph I	Ongoing	NantKwest Inc, USA
	Not available	CD19/CD22	unknown	PB-NK	B-ALL	Ph I	Planned	Avalon GloboCare Corp, China
	Not available	CD19/CD22	unknown	PB-NK	Non-Hodgkin Lymphoma	Ph I	Planned	Avalon GloboCare Corp, China
	Not available	EpCAM	unknown	NK101 cell line	Ovarian Cancer	Ph I	Planned	SL Bigen Inc, South Korea
	Not available	IL13Rα	unknown	NK101 cell line	Glioblastoma	Ph I	Planned	SL Bigen Inc, South Korea
	Not available	FLT3	unknown	NK101 cell line	AML	Ph I	Planned	SL Bigen Inc, South Korea
	Not available	BCMA (GoCAR-NK)	IL-15	Primary NK cells	Multiple Myeloma	Preclinical	Planned	Bellicum Phamaceuticals
	NCT02944162	CD33	None	NK-92 cell line	AML	Ph I	Ongoing, recruiting	PersonGen Biomedicine Suzhou Co Ltd, China
	NCT03941457	ROBO1 (BiCAR)	None	NK-92 cell line	Metastatic Pancreatic Cancer	Ph I/II	Ongoing, recruiting	Asclepius Technology Company Group Suzhou Co Ltd, China
	NCT03940833	BCMA	None	NK-92 cell line	Multiple Myeloma	Ph I/II	Ongoing, recruiting	Asclepius Technology Company Group Suzhou Co Ltd, China
	NCT03940820	ROBO1	None	NK/T	Malignant Neoplasms	Ph I/II	Ongoing, recruiting	Asclepius Technology Company Group Suzhou Co Ltd, China
	NCT02892695	CD19	None	NK-92 cell line	Leukemia	Ph I/II	Planned	PersonGen Biomedicine Suzhou Co Ltd, China
	NCT02742727	CD7	None	NK-92 cell line	AML, T-cell leukemia, lymphoma	Ph I/II	Recruiting	PersonGen BioTherapeutics (Suzhou) Co., Ltd, China
	NCT02839954	MUC1	None	NK-92 cell line	HCC, NSCLC, pancreatic carcinoma, TN breast cancer, glioma, CRC, gastric carcinoma	Ph I/II	Recruiting	PersonGen BioTherapeutics (Suzhou) Co., Ltd, China
	NCT04639739	CD19	None	NK cell	relapsed and refractory B cell non-Hodgkin lymphoma	Ph 0	Planned	Xinqiao Hospital, Chongqing, China
Academic	NCT03056339	CD19	chemotherapy + BSCT	CB-NK	B cell lymphoma or leukemia	Ph I/II	Ongoing	M.D. Anderson Cancer Center, USA
	NCT03383978	HER2	None	NK-92 cell line	Glioblastoma	Ph I/II	Ongoing	Johann W. Goethe University Hospital, Germany
	NCT01974479	CD19	IL-2 sc	PB-NK on K562-mbIL15-41BBL	ALL	Ph I/II	Suspended for an interim review	National University Health System, Singapore

*PB-NK* peripheral blood-derived natural killer cells, *CB-NK* cord blood-derived natural killer cells

The treatment was safe as no CRS, neurotoxicity or GVHD occurred. From the 11 treated patients, 8 responded with 7 patients going into complete remission, although 3 patients had minimal residual disease at final assessment. CAR-NK cells were tracked in vivo and could be detected rather stable over time and for at least 12 months. It was suggested that in vivo persistence was driven by proliferation of the CAR-NK cells, which could be caused by the addition of IL-15 in the CAR construct (hence one could define this as a fourth-generation CAR (see section Activation signal for CAR-NK and Table 3). At least in mice, it has been shown that IL-15 aids in the persistence of (dual-switch CD123 or BCMA) CAR-NK cells for minimal 40–50 days [69], when the experiments were stopped.

In addition, the first patient treated with iPSC-derived CAR-NK cells has recently been reported, also suggesting that CAR-NK cells can outperform CAR-T cells [28, 164]. Still, the clinical trial is ongoing and the results have to be awaited. While many questions regarding CAR-NK cell therapy in humans remain, the large number of preclinical investigations imply that it is only a matter of time before more clinical trials are conducted.

### Technological advances in CAR-NK generation: biomaterials and in vivo transduction

CAR-NK development was built on the knowledge gained from the CAR-T field. To further the development of CAR-NK for clinical use, technological advances will importantly speed up the process. Here, we consider two developments that will aid in the generation of sufficient CAR-NK cells for clinical use and for the in vivo generation of CAR-NK cells.

Biomaterials have shown promise as a concomitant agent together with effector cells for immunotherapy that can aid in mounting a stronger response, while simultaneously lowering the risk for side effects [165]. In NK cells, biomaterial micelles are able to facilitate the formation of an immune synapse with tumor cells, aiding in the eradication of solid malignant lumps in vivo [166]. A three-dimensional engineered hyaluronic acid-based niche (3D-ENHANCE), instead of the classic two-dimensional culture methods, was shown to promote CAR-NK cell expansion and to enhance cytokine release, while maintaining killing capacity of EGFR-CAR-NK92 towards solid tumors in vivo [167]. In another study, biomaterial-modified Fc fragments on antibodies showed a strong potential to broaden NK cell recognition of heterogeneous antigens on solid tumors [168]. Moreover, Smit et al. reported that DNA-carrying nanoparticles can efficiently introduce leukemia-targeting CAR genes into T-cells [169], which was also reported for NK-92MI cells in vitro [170]. Thus, various promising inventions from the bio-engineering field are likely to enter the CAR field as important improvements in the design and engineering of cancer immunotherapy.

Various groups are trying to harness autologous lymphocytes, including NK cells for immunotherapy without the need for ex vivo modifications [171]. This presents various challenges, such as achieving proper activation of NK cells without haplotype-dependent, allogeneic activation. Furthermore, in vivo proliferation and maintenance is required to obtain sufficient cell numbers to mount a successful anti-tumor response. Recently, it has been shown that the engineering of the glycoprotein on the VSV-G virus with anti-CD8 scFV makes it possible to specifically transduce CD8 T cells in vivo [171]. Using this concept, Pfeiffer et al. showed another approach of in vivo CAR-T generation by injecting lentivirus directly into laboratory mice. Indeed, in vivo injection of the CD8 specific-CD19 CAR-encoding lentiviruses resulted in transduction solely of the CD8^+^ T cells with a CD19-CAR [172]. However, this method also led to signs of a CRS, thus warranting further optimization before this in vivo CAR-T generation concept could be implemented in the clinic. These methods have not been tried on NK cells; we envisage that the concepts of these techniques can easily be applied to CAR-NK cell generation as well.

### Challenges

Still many aspects on CAR constructs will have to be improved and many challenges lie ahead of the field. Besides the constructs, other aspects have to be taken into account. Here, we discuss three important challenges for the CAR-NK field that will need to be addressed in the near future regarding clinical safety, cryopreservation of CAR-NK cells and challenges posed by the tumor microenvironment.

The potential of CAR-based therapy has been clearly demonstrated, but it is not without risks. Indeed, patients treated with CAR-T cells have died because of cytokine release syndrome and off-target cytotoxic effects [1, 173]. While improvements in CAR design have made CAR-based therapy safer by reducing these severe side effects [31, 174], the incorporation of safety switches that quickly eliminate the CAR-NK infused cells, provides another level of safety [69, 175]. CAR-NK cells are in general considered safer than CAR-T cells, although there is very limited clinical experience with CAR-NK therapy in patients, justifying additional safety measures, including the use of “suicide genes” [117].

Indeed, inducible caspase 9 (iCasp9) was included as a safety switch in the first reported clinical trial of CAR-NK [30]. While no severe side effects warranting the need to use this safety switch were reported, it might be necessary to use it at a later time point, since CAR-NK cells were still detected after one year in vivo. The inducible safety switch caspase 9 (iCasp9) suicide gene is a modified caspase 9 gene fused to the human FK506 binding protein gene (FKBP). Addition of the inducer of dimerization (CID) (AP1903) leads to dimerization and recruits the downstream caspase molecules, thereby initiating apoptosis [176].

Recently, Ruella et al. demonstrated that anti-CAR19 idiotype chimeric antigen receptor (aCAR19) cells could eliminate CAR19^+^ T cells [177]. This CAR-T eliminating other CAR-T cells is not only useful for eliminating infused cells in the case of side effects, but also at the same time it depletes all transduced cells (including leukemic blasts) present during CAR-T manufacturing, minimizing the potential clinical effect, but preventing side effects. Additionally, these cells can be used to deplete CAR-T cells to reduce long-term side effects, such as B cell aplasia in the case of CD19-targeting CARs.

Truncated EGFR [178], CD19, CD20 or CD34 epitopes together with the CAR gene have been developed for CAR-T therapies [179]. Once the CAR-transduced cells cause undesirable effects, an additional administration of a specific antibody against these epitopes will induce ADCC to eliminate the CAR-NK cells in vivo [29, 69, 180, 181].

Not all issues related to the use of lentivirus for clinical use have already been resolved. For instance, it has been demonstrated that lentivirus-mediated p53 activation can lead to delayed cell proliferation, cell cycle arrest and slightly but significantly increased apoptosis during cell culture [182]. In a study using autologous CAR-T manufacturing, it was reported that the CAR gene was inadvertently transduced into a single leukemic B cell. The anti-CD19-scFv combined with the CD19 molecule on the surface of all leukemic B cells derived from this clone, camouflaging it from recognition by the CAR-T and becoming insensitive to the aimed effect [183]. Another case illustrated a patient suffering from chronic lymphocytic leukemia, in which after infusion with engineered autologous T cells, 94% of the CAR-T cells were found to originate from a single clone in which the lentiviral vector with the CAR transgene disrupted the methylcytosine dioxygenase *TET2* gene [184]. Fortunately, the *TET2* disruption by the lentivirus integration altered the epigenetic profile of the T cells that resulted in enhanced differentiation and expansion and exerted a central memory phenotype [184].

Retrovirus-mediated gene transduction has been reported to lead to random insertions creating the risk of leukemia [185, 186]. However, with advances in the development of viral vectors and detection technology, June’s group published 17 vector lots, 375 manufactured T cell products, and 308 treated patients with both oncologic and HIV-related indications for treatment (2001–2016), showing no evidence of generation of replication-competent retroviruses or lentiviruses [187].

Genome editing by CRISPR–Cas9 has been reported to induce p53-mediated DNA damage responses and cell cycle arrest in human cells [188]. Moreover, the potential of pre-existing humoral and cell-mediated adaptive immune responses to the Cas9 system in humans should be taken seriously when this system is applied in vivo during clinical trials [189]. All these issues will have to be addressed to guarantee the safe use of CAR-T and CAR-NK in patients.

An important technical issue is the cryopreservation of NK cell. For clinical application and infusion of cells into patients, large-scale expansion of CAR-NK cells under good manufacturing practice (GMP) is required by authorities. It is hardly possible that a freshly expanded CAR-NK cell product can be administered into patients at a suitable time point taking into account the status of the cells and the logistics and timing to prepare the patient [162]. Cryopreservation of CAR-NK cells is a necessity, but also allows to use NK cells as “off-the-shelf” products. This measure should guarantee the recipient always receives a similar quality of CAR-NK cells, thus disregarding the natural variety in NK cell donors [190]. Numerous groups are trying to optimize the ingredients of the freezing medium and procedures for expanded CAR-NK cells and have achieved encouraging outcomes [162, 190, 191]. A recent approach uses nanoparticle-mediated intracellular protection of NK cells which can avoid cryoinjury and maintain the killing potential of NK-92 cells, which could completely replace DMSO as a freezing protector [192]. This technique still has to be tried on and optimized for primary NK cells.

Even if the best CAR-NK cells can be made, they always will encounter the suppressive tumor microenvironment, possibly the biggest challenge of all to overcome [193]. So far, much effort is put in to designing CARs or combinations of treatments in overcoming the negative effects of the TME. CARs have been combined with checkpoint inhibitors like PD-1 [194, 195] and were successful in mice [194], but less in human [195]. Depletion of soluble suppressive factors like TGF-β, IDO and IL-4 can be obtained by expressing of dominant negative mutants or gene editing of the respective genes [196, 197]. Many solid tumors are characterized by hypoxic cores which may influence the function of NK cells, or on the other hand using and an HIF1α-driven CAR one could take advantage of the hypoxic environment [198]. A reduction of immune suppressor cells that are abundantly present in the TME (Treg [199], MDSC [200], TAM [201]) also is directed towards an improved clinical outcome and increased overall survival. The challenges posed by the tumor microenvironment and possible solutions using CAR-engineered cells have been covered extensively in various recent reviews [193, 202].

## Conclusion

In this review, we performed a comprehensive analysis of preclinical and clinical studies from the CAR-NK field with an emphasis on the design of the CAR and engineering of NK cells. Recommendations for usage of gene elements have been described, as well as the state of the art, including important lessons from CAR-T development. This detailed information will pave the way to more robust CAR-NK cells studies with the aim of getting a stronger anti-tumor response with fewer side effects compared to CAR-T cells. Overcoming the suppressive tumor microenvironment remains an important challenge on the design of CARs before CAR-NK cells, in combination with other therapies, will fulfill their promise.

## Supplementary Information

**Additional file 1. Table S1:** Cell line-derived CAR-NK cells (extended version)

**Additional file 2. Table S2:** Primary cell-derived CAR-NK cells (extended version)

## Data Availability

The interactive tables for CAR-NK design are on www.carnkreview.com. All data generated or analyzed during this study are included in this published article (and its supplementary information files).

## Abbreviations

ADCC: Antibody-dependent cellular cytotoxicity
ALL: Acute lymphoblastic leukemia
AML: Acute myeloid leukemia
APC: Antigen-presenting cell
BCMA: B cell maturation antigen
CAR: Chimeric antigen receptor
Cas9: CRISPR-associated system 9
CCR: CC chemokine receptor
CD: Cluster of differentiation
CLL: Chronic lymphocytic leukemia
CR: Complete remission
CRS: Cytokine release syndrome
CRISPR: Clustered regularly interspaced short palindromic repeats
DLBCL: Diffuse large B cell lymphoma
EGFR: Epidermal growth factor receptor
FasL: Fas ligand
FDA: US Food and Drug Administration
GM-CSF: Granulocyte-macrophage colony-stimulating factor
GVHD: Graft-versus-host disease
Her2: Human epidermal growth factor receptor 2
HLA: Human leukocyte antigen
HSPC: Hematopoietic stem/progenitor cell
IFN-γ: Interferon-γ
iPSC: Induced pluripotent stem cell
IS: Immunologic synapse
ITAM: Immunoreceptor tyrosine-based activation motif
KIR: Killer immunoglobulin receptor
MHC: Major histocompatibility complex
MoAb: Monoclonal antibody
MM: Multiple myeloma
MOI: Multiplicity of infection
NCR: Natural cytotoxicity receptor
NK: Natural killer
NSG: NOD scid gamma
PB: Peripheral blood
PR: Partial remission
PSMA: Prostate-specific membrane antigen
PSCA: Prostate stem cell antigen
RT-PCR: Real-time PCR
scFv: Single-chain antibody variable fragment
TAA: Tumor-associated antigen
TCR: T cell receptor
TGF-β: Transforming growth factor-beta
TME: Tumor microenvironment
TRAIL: TNF-related apoptosis-inducing ligand
UCB: Umbilical cord blood
